# Supplementary material for: Mapping the Global Chromatin Connectivity Network for Sox2 Function in Neural Stem Cell Maintenance
Source: Cell Stem Cell. 2019 Mar 7;24(3):462–476.e6. doi: 10.1016/j.stem.2019.02.004 (PMC6506828; doi:10.1016/j.stem.2019.02.004)
Supplement: Document S1. Figures S1–S6 and Tables S2, S6, and S7 [file mmc1.pdf]

Cell Stem Cell, Volume *24*

Supplemental Information

Mapping the Global Chromatin Connectivity Network for Sox2 Function in Neural Stem Cell Maintenance

Jessica A. Bertolini, Rebecca Favaro, Yanfen Zhu, Miriam Pagin, Chew Yee Ngan, Chee Hong Wong, Harianto Tjong, Marit W. Vermunt, Ben Martynoga, Cristiana Barone, Jessica Mariani, Marcos Julián Cardozo, Noemi Tabanera, Federico Zambelli, Sara Mercurio, Sergio Ottolenghi, Paul Robson, Menno P. Creyghton, Paola Bovolenta, Giulio Pavesi, Francois Guillemot, Silvia K. Nicolis, and Chia-Lin Wei

Supplemental Information

Figures S1-S6
Tables S2, S6, S7; Tables S1, S3, S4, S5, S8 are separate Excel files.

Figure S1, related to Figure 1
Overview of Chia-PET and in situ ChIA-PET workflow

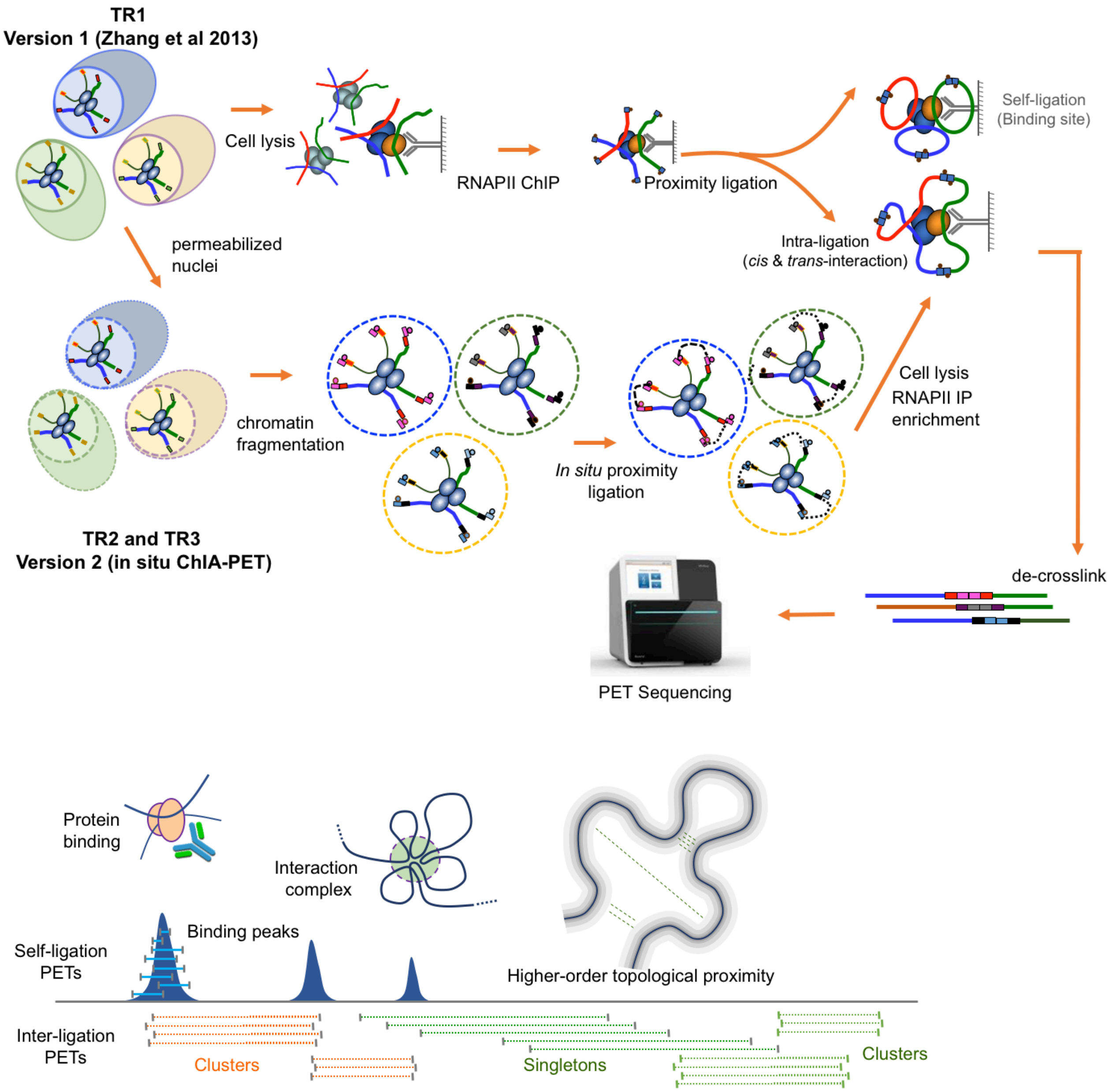

ChIA-PET was as previously described by Zhang et al., 2013; after crosslinking, all additional procedures were performed after cell lysis. For in situ ChIA-PET, the nuclei of the crosslinked cells were permeabilized when still intact, allowing to process chromatin for proximity ligation prior to cell lysis (see Methods).
In the bottom panel, different types of inter-ligation PETs are shown. In addition to revealing RNAPolII-mediated interactions, in situ ChIA-PET may reveal, when considering the entire set of intra-molecular ligated PETs (bottom panel) also general chromatin contacts that represent broad spatial topological associated domains, similar to the global contact maps generated by Hi-C based approaches (data not shown).

Figure S2, related to Figure 1
Examples of reproducibility in different biological replicates

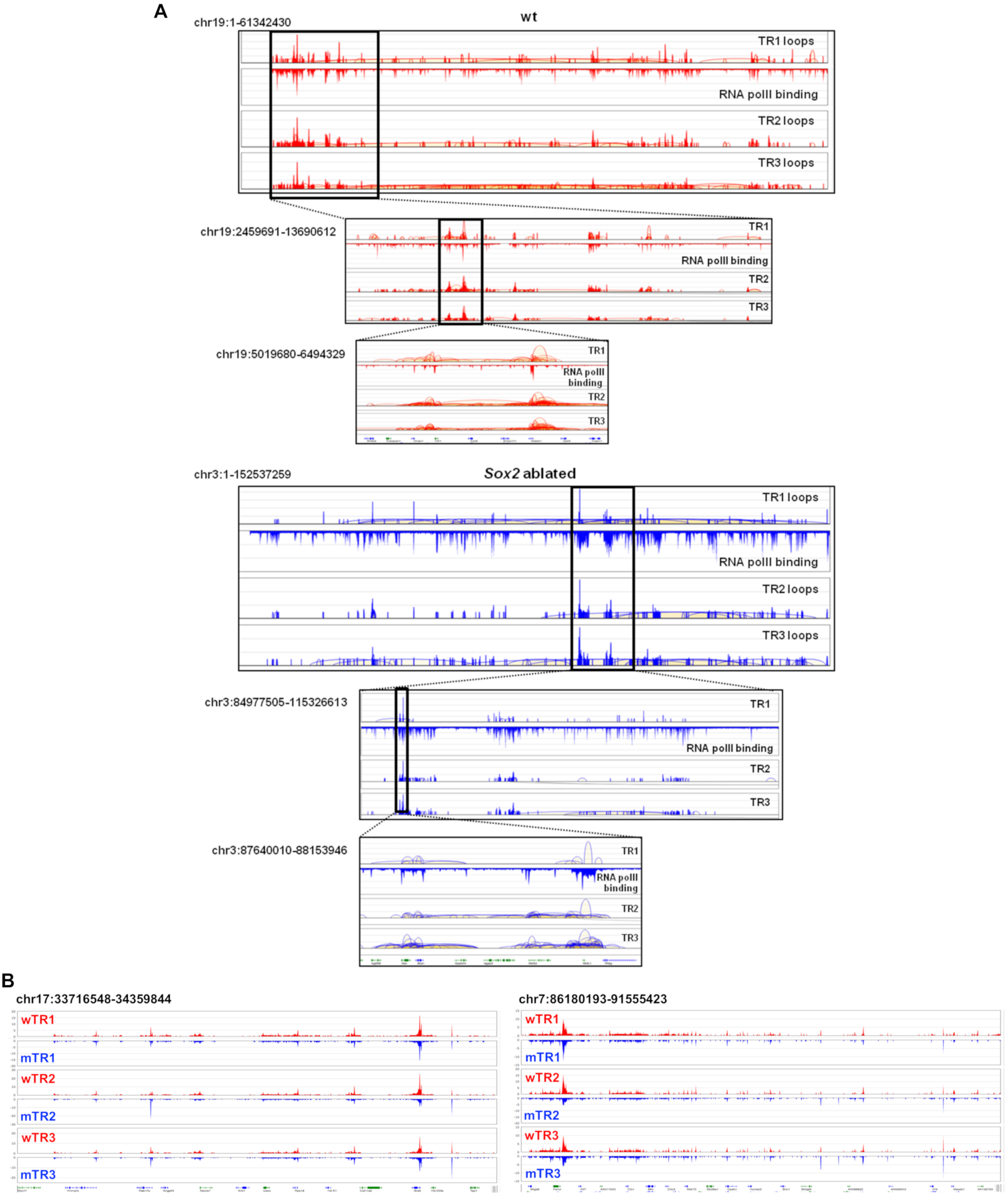

(A) Examples of interactions (loops) profiles in TR1, TR3, TR3, in wild-type cells (red, top) and mutant cells (blue, bottom), in the indicated chromosome regions. The boxed regions are zoomed-in to allow observation at higher resolution.

(B) Examples of RNApolII binding profiles in wild type and mutant TR1, TR2 and TR3 samples.

Figure S3, related to Figure 1
Comparison of PET counts between wild-type and Sox2-mutant libraries (TR2 and TR3) across 9 view points

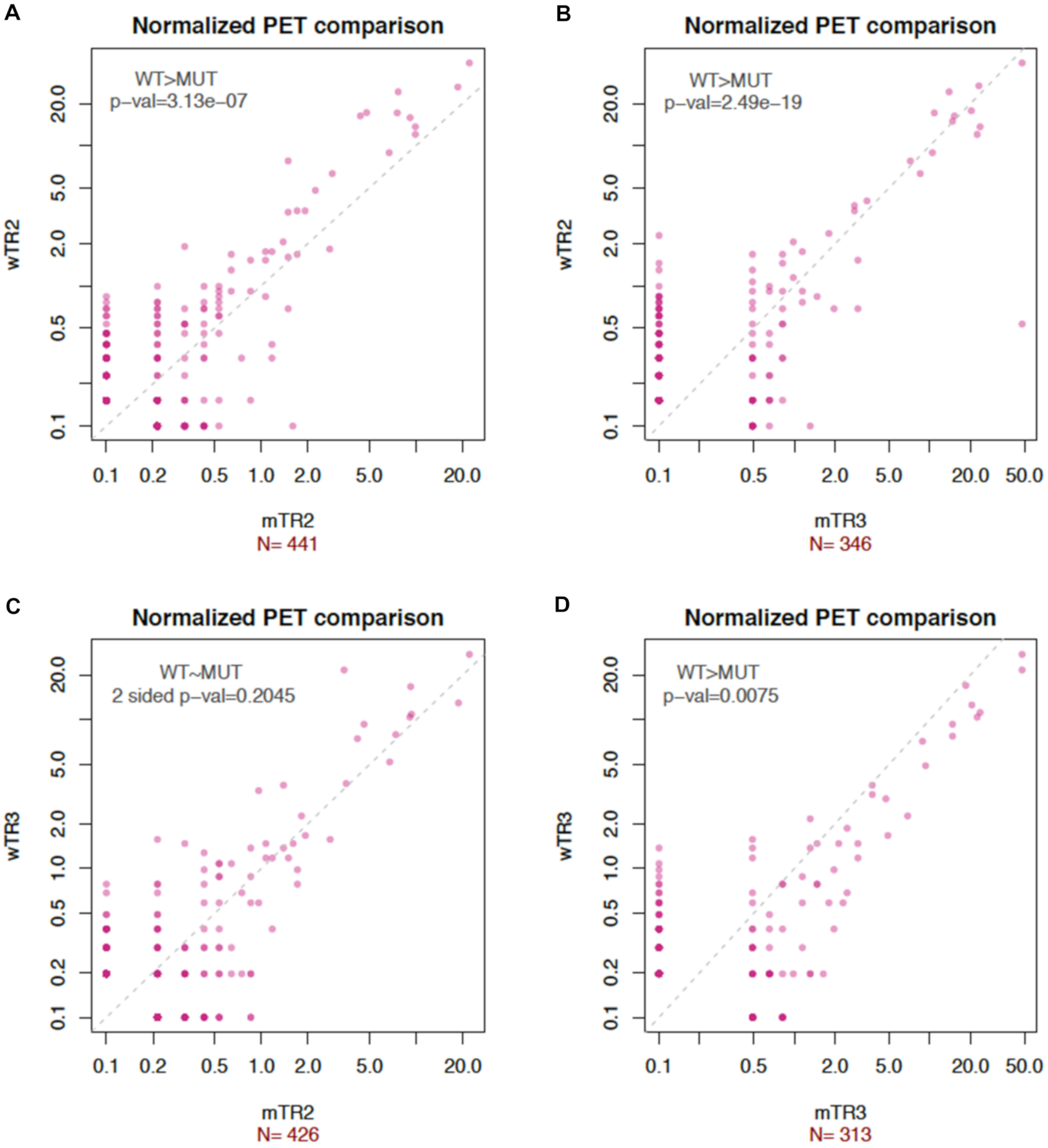

Comparisons are:
A) between wtTR2 and mtTR2; B) between wtTR2 and mtTR3;
C) between wtTR3 and mtTR2; D) between wtTR3 and mtTR3. All cis-interactions along the chromosome from compared libraries that originated from all view points listed in the Table below (+/- 5kb) were collected. The interaction anchors were then evaluated to assess whether they overlap. As a result, a new set of non-overlapping interactions was then collected (see the Table for number of interactions or dots on each panel). When an interaction was absent in one of the two libraries, its PET count was assigned as 0. The compared data set is compiled from the output of a significance-calling algorithm, using down-sampled data (Table 1) to minimize sequencing depth bias.

The PET counts for each interaction (normalized by dividing by the total number of intrachromosomal PETs -Table 1, line 6- , then multiplying by one million) are plotted as dots, with WT PET count on the Y axis, and MUT PET count on the X axis; the dashed line represents equal interaction frequency, so points above this line indicate reduction of interaction frequency in MUT. Using log-log scale plotting, the zero PET counts are all augmented by 0.1. Because the dots are transparent, the color intensity of the dots indicates multiple dots having the same WT/MUT PET counts. We statistically evaluated differences between wild type and mutant; differences for MUT < WT are shown as p-values on the plot area (2-sided Wilcoxon rank sum test p-values). mTR3 was highly significantly reduced relative to wTR2 and wTR3; mTR2 was less significantly reduced. The comparison of WT and MUT replicates between themselves yielded non-significant p-values (0.029 and 0.09039, respectively). For this analysis, the threshold for interaction calling was set to PET count = 2, FDR < 0,05 across all 9 loci, yielding a number of interactions adequate for statistical analysis. N: the total number of interactions (dots on the plots) evaluated for each comparison.
The numbers of PET counts are listed in Table S3.

For each viewpoint, the genomic coordinates are given. Number of interactions (dots) are shown on the right for each viewpoint, and each panel.

	**Viewpoint**	**CHR**	**Mid_Coordinate**	**panel A**	**panel B**	**panel C**	**panel D**
1	c-Fos-enh5	chr12	86,797,329	43	36	39	32
2	c-Fos-prom	chr12	86,814,992	63	53	70	58
3	c-Fos_enh3	chr12	86,829,043	54	47	38	29
4	Sox3_prom	chrX	58,145,388	10	7	11	8
5	Olig1_prom	chr16	91,270,745	66	54	74	53
6	Olig2_prom	chr16	91,224,410	102	80	93	70
7	Kat2b_prom	chr17	53,707,872	24	20	20	13
8	Tcf4_prom	chr18	69,505,556	29	10	34	17
9	Stox2_prom	chr8	48,372,611	50	39	47	33
**Total**				**441**	**346**	**426**	**313**

Figure S4, related to Figure 2

Long-range interaction anchors are enriched in SOX2 binding and in chromatin epigenetic marks of active/poised enhancers

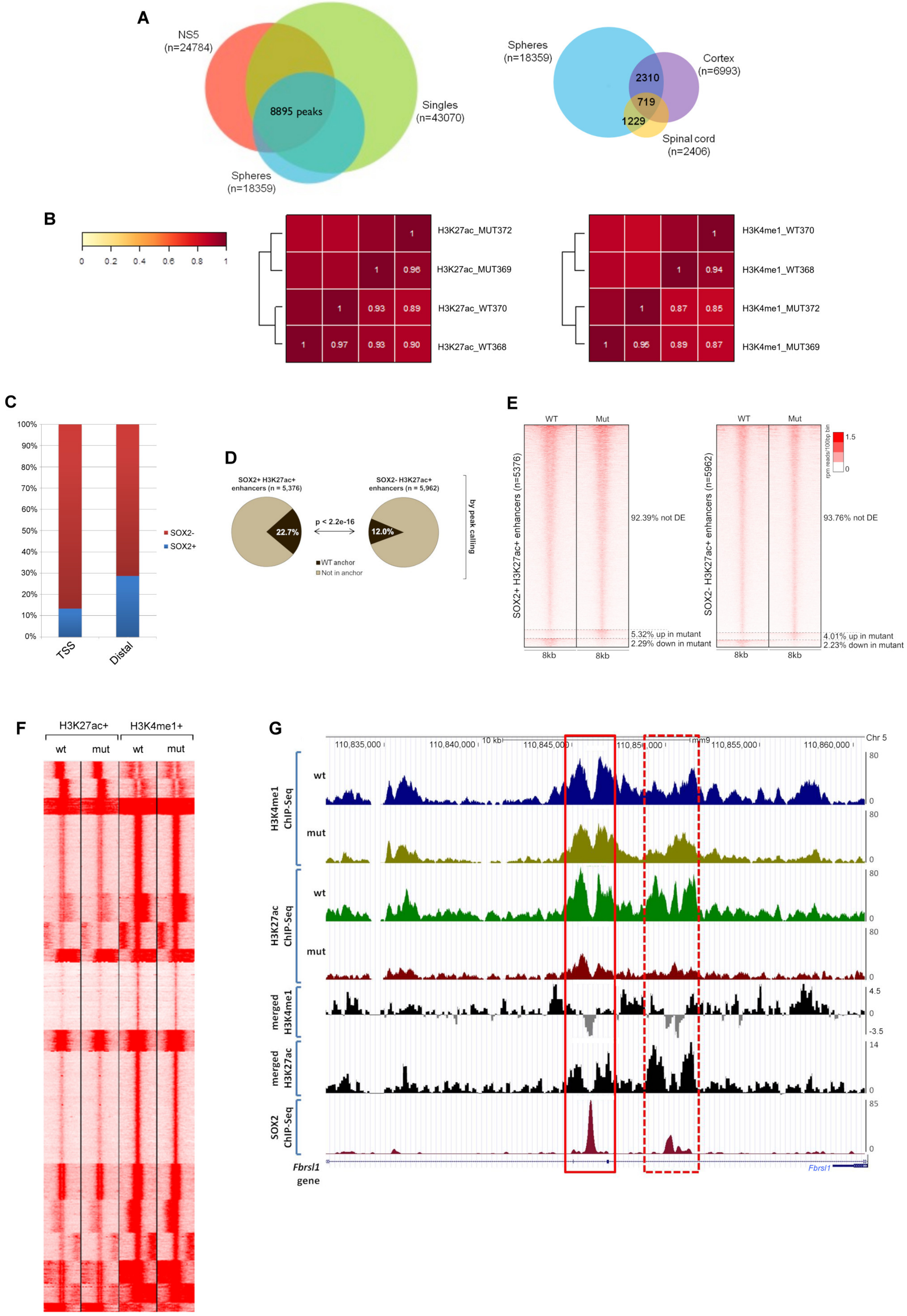

(A) SOX2 ChIPseq was performed on both intact ("spheres") and dissociated ("singles") neurospheres (see Methods). Ca. 87% of peaks identified in spheres are also present in singles; ca. 50% of peaks in spheres overlap with peaks in the ES cell-derived NS-5 neural stem cell line (Mateo et al., 2015). Ca. 33% and 51% of peaks detected in neural progenitors of cortical and spinal cord origin, and 68% of their intersection (Hagey et al., 2016), are also detected in spheres.
(B) Pearson correlations between the samples for each antibody used in duplicate samples.
(C) Fraction of SOX2+ and SOX2- regions within epigenetically marked regions (i.e. H3K27ac+ and/or H3K4me1+) (ChromHMM).
(D) Fraction of Sox2+ vs. Sox2- EM-positive regions which overlap with anchors. Distal (>1000nt from TSS) Ac+ regions, peak-calling.
(E) *Sox2* loss does not result in H3K27ac-enrichment changes at enhancers. Heatmap depicts H3K27ac enrichment (for one wt and one mutant line) for SOX2-positive and SOX2-negative enhancers. Reads were counted within 40 bins of 100 bps up- and downstream of the enhancer centre. The fraction of (not) significantly differentially enriched enhancers, as defined using DESeq2, is indicated.
(F) *Sox2* loss does not result in H3K27ac- or H3K4me1-enrichment changes at regions bound by SOX2 in wt cells.
(G) A representative example of quantitative differences in epigenetic enhancer marks between wt and mut cells (SOX2 peaks within the *Fbrsl1/AUTS2l* gene, boxed in red). Merged profiles represent the variation of enrichment, positive values correspond to greater enrichment in wt, negative in mut, respectively. Typically, H3K27ac has two peaks flanking the SOX2 binding site: the height of these peaks is decreased in mutant cells, whereas H3K4me1 has a moderate increase in mut closer to SOX2 binding sites, as also shown for the second SOX2 peak on the right, where similar changes occur.

Figure S5, related to Fig. 3
Enrichment of VISTA enhancers within interaction anchors

A

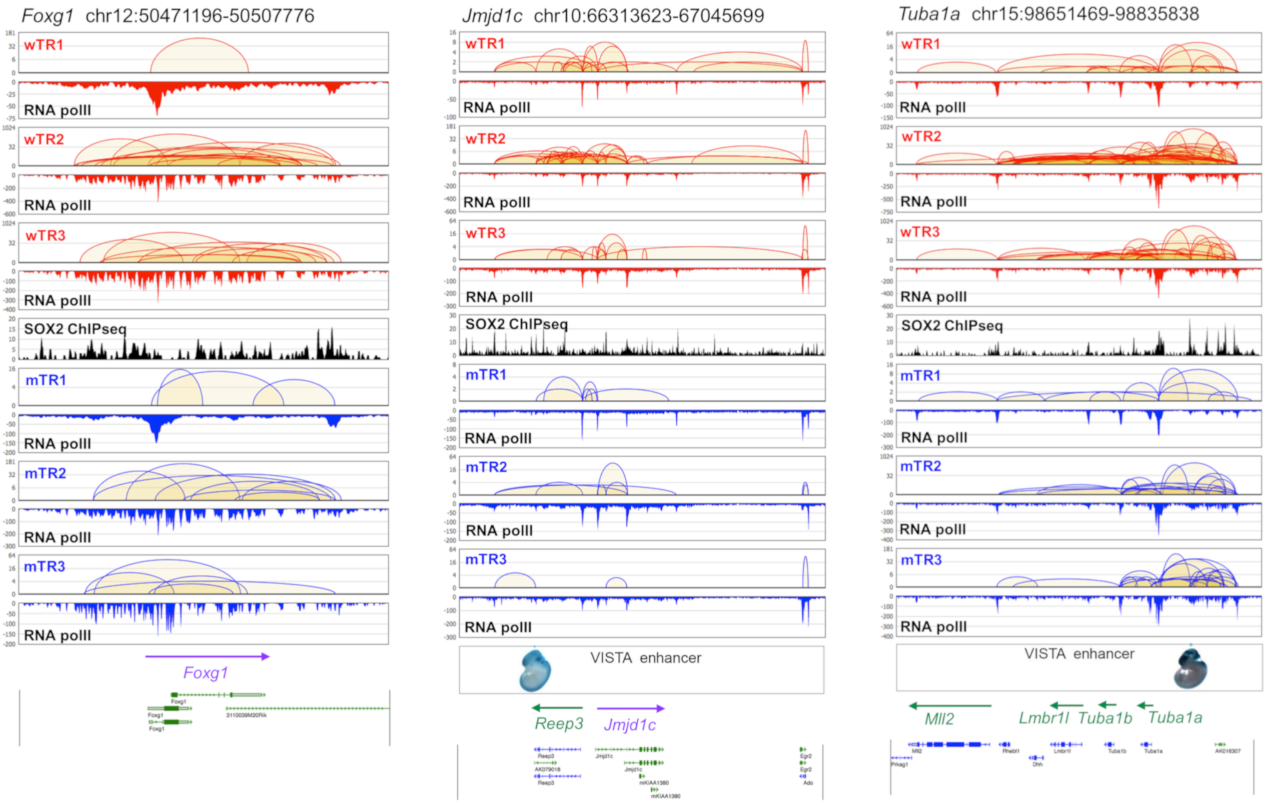

B

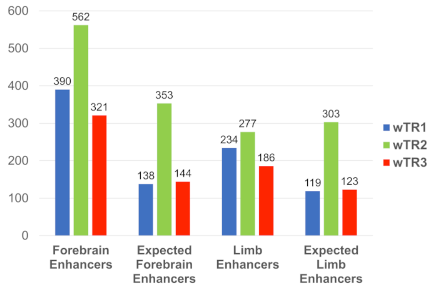

Forebrain p300 enhancers	Total number	2454

	VISTA enhancers in all anchors	VISTA enhancers in distal anchors	% Overlap of VISTA enhancers with anchors	% Distal overlap*	No. of anchors	No. of distal anchors	% of distal anchors	Expected No. of overlapping VISTA enhancers in anchors
wTR1	390	256	15.89	65.64	14138	4639	32.81	138
wTR2	562	405	22.90	72.06	36012	13435	37.31	353
wTR3	321	211	13.08	65.73	14680	5005	34.09	144
mTR1	104	31	4.24	29.81	6488	2404	37.05	63
mTR2	105	33	4.28	31.43	5742	1681	29.28	56
mTR3	99	35	4.03	35.35	6402	2234	34.90	62

* % distal versus total VISTA enhancers overlapping with anchors

(A) Examples of replicates of ChIA-PET analysis of regions surrounding genes encoding proteins important for inherited brain developmental defects (See also **Table S7**). Images (from https://enhancer.lbl.gov/) of embryos carrying lacZ transgenes driven by VISTA enhancers located within the considered genomic regions are shown.
(B) Left: Numbers of VISTA enhancers from forebrain or limb (forebrain enhancers, limb enhancers; Visel et al., 2009) in interaction anchors, as compared to the numbers expected based on a random distribution. Limb enhancers are presented as a term of comparison between regulatory elements active in forebrain neural tissue versus non-neural tissue.
Right: Overlap of forebrain VISTA enhancers with anchor types.

Figure S6, related to Figures 3 and 4
Regulation of the Sp8 enhancer-dependent GFP-reporter construct by decreased or increased Sox2 expression

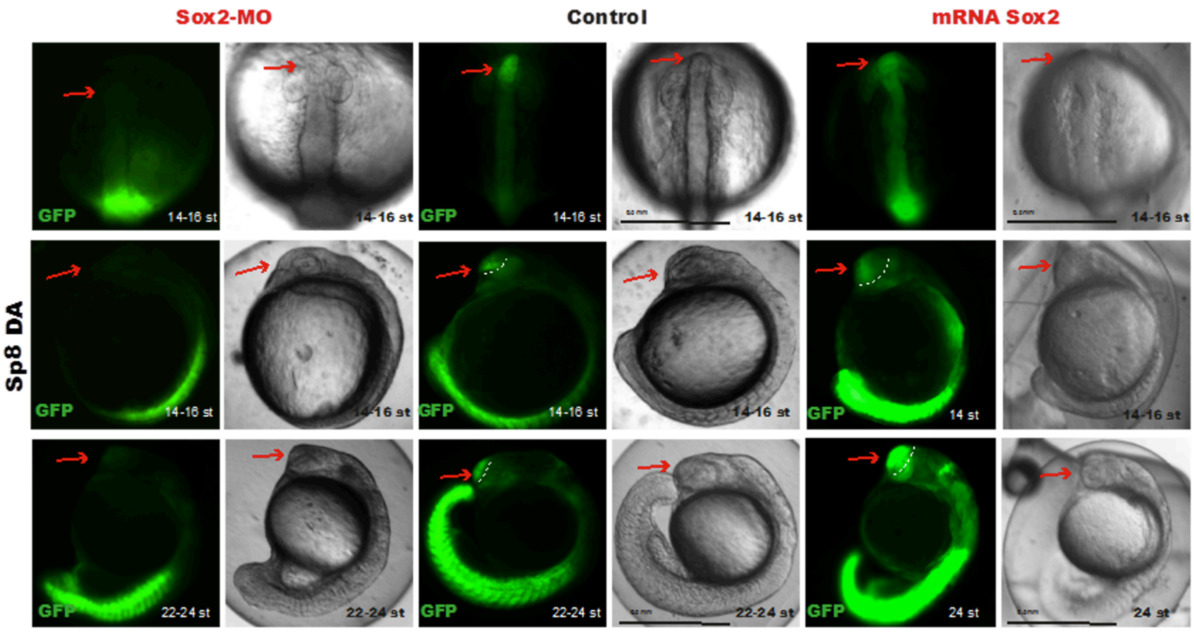

Transgenic embryos injected with anti-Sox2 morpholino (*Sox2*-MO, left), control morpholino (center), Sox2mRNA and analyzed at two different stages. Reduced GFP signal is seen in *Sox2*-MO, but not ctrl-MO embryos, in forebrain (red arrow) but not in more posterior regions (internal control). Sox2 mRNA extends the forebrain expression of the transgene (in 19/54 embryos). Top, dorsal view; middle and bottom, lateral view.

Tables S2, S6, S7

(Excel Supplemental Tables are provided separately)

Table S2, related to Figure 1
Average reproducibility score (SCC, Stratum-adjusted Correlation Coefficient*) between TR2 & TR3 over all 20 chromosomes
WT: 0.935 (wTR2 vs. wTR3).
MUT: 0.839 (mutTR2 vs. mutTR3).

SCC break down by chromosome between replicas:

**Chrom**	WT	MUT
chr1	0.938	0.846
chr2	0.918	0.813
chr3	0.935	0.819
chr4	0.946	0.859
chr5	0.920	0.826
chr6	0.920	0.815
chr7	0.944	0.857
chr8	0.936	0.854
chr9	0.930	0.827
chr10	0.925	0.813
chr11	0.924	0.823
chr12	0.946	0.866
chr13	0.964	0.877
chr14	0.951	0.867
chr15	0.933	0.849
chr16	0.941	0.850
chr17	0.939	0.862
chr18	0.944	0.855
chr19	0.942	0.855
chrX	0.905	0.755

Method:
To compute the SCC, ChiA-PET loops for each library were aggregated into 10-kb bin matrix.
SCC score is computed with HiCrep tool (hicrep library in R; Yang et al. (2017) Genome Research 27:1939).
For each chromosome, the smoothing parameter used as recommended (h=5) and maximum distance 1 Mb.

*reference: https://genome.cshlp.org/content/early/2017/08/30/gr.220640.117
Pearson's correlation coefficient (PCC) is computed for loops within the same genomic distance. From all distances considered, the PCCs are then weight averaged.

Table S6, related to Figure 3, Fig. S5 and Table S5
Interactions and SOX2 peaks in mouse homologs of genes involved in inherited neural disease in man

Category of disease and gene name in mouse	Disease gene promoter interaction with distal enhancer: wt-sp	wt-alt	com	SOX2-bound enh.	SOX2-bound
**Microcephaly associated to defects in**					
**centrosome and spindle microtubule (1)**					
positive: 10/22; p-value: 0.006					
Cdk5rap2 (Mcph3)					•
Casc5					•
Cenpj					•
Stil					•
Cep63		•		•	
Kif2a		•			
Kif11	•				
Tubb2b		•	•	•	•
Tuba1a		•	•	•	•
Poc1a					•
**origin recognition complex core (1)**					
positive: 2/5					
Orc4					•
Cdt1					•
**DNA damage response and repair (1)**					
positive: 5/19					
Lig4					•
Phc1 (Mcph11)		•		•	•
Xrcc2					•
Xrcc4					•
Blm (Recql3)					•
**Other microcephalies**					
Gpr56		•	•	•	•
Cdk19					•
Arx			•		
Zbtb18					•
**Angelman and Angelman-like syndromes (2)**					
**(intellectual disability and absent speech)**					
positive: 10/12; p-value: 0.0000065					
Ube3a					•
Tcf4	•	•	•	•	•
Ehmt1					•
Herc2		•		•	
Adsl					•
Cdkl5					•
MeCP2					•
Foxg1		•		•	
Atrx					•
Zeb2	•	•		•	•

Category of disease and gene name in mouse	Disease gene promoter interaction with distal enhancer: wt-sp	wt-alt	com	SOX2-bound enh.	SOX2-bound
**Histone modification, chromatin remodelling**					
**and mediator mutations (3,4,5)**					
**(intellectual disability)**					
positive: 8/12; p-value: 0.0007					
Med17					•
Med23					•
Med25					•
Smarca2					•
Arid1a		•	•	•	
Arid1b		•	•		
Jmjd1c		•	•	•	
Phf21a					•
**Cohesin subunit mutations (6)**					
**(psicomotor delay, intellectual disability)**					
positive: 4/14					
Smc3					•
Rad21 (Scc1)					•
Stag1		•		•	
Stag2		•			
**Microphthalmia / Anopththalmia /**					
**Coloboma and other eye pathologies (7)**					
Otx2		•			•
Pax6					•
Six3					•
Bmp7	•			•	
Grcc10 (C12orf57)		•			•
Sall2					•
RarB	•				•
Smoc1	•			•	
Wdr19					•
COUP-TF1 (Nr2f1)	•			•	
Abhd12	•			•	

References:
(1) Alcantara and O'Driscoll, 2014, Am J Med Genet C Semin Med Genet 166C, 124
(2) Tan et al., 2014, Am J Med Genet A 164°, 975-992
(3) Lee and Young, 2013, Cell 152, 1237-1251
(4) Saez et al., 2016, Genetics in Medicine 18, 378-385
(5) Kim et al., 2012, Am J Hum Genet 91, 56-72
(6) Peters et al., 2008, Genes Dev 22, 3089-3114
(7) Williamson and Fitzpatrick, 2014, Eur J Med Genet 57, 369-380

We list genes related to the main categories of neurodevelopmental disorders in man that are either present in SOX2-mutant patients (hippocampal, eye defects; intellectual disability, seizures) and/or in Sox2-mutant mice (microcephaly, hippocampal defects, seizures, eye defects). Disease genes are analyzed for interactions of the respective mouse gene promoter with distal enhancers in wTR1, or presence of SOX2. For replicates (wTR2 and wTR3) of connectivity analyses for some of these genes see Fig. 3 and Fig. S5. p-values are given, when significant, for the association between the above characteristics and type of disease.

Table S7, related to Figure 4
Summary of transgenic experiments in zebrafish

Gene associated with the proximal anchor	Cloned anchor [DA: distal anchor; PA: proximal anchor]	Distance PA-DA [bp]	Presence of SOX2 peaks (ChIP-seq)				Presence of already defined forebrain enhancer (VISTA enhancer)		Tested in fish (GFP+ at 24 hpf)				
			cloned anchor		associated anchor		p300 binding site	validated enhancer	reproducible GFP+ expression in forebrain	GFP+ in forebrain / tot transgenics		GFP+ in forebrain and elsewhere / tot transgenics	intensity of GFP+ expression in forebrain
			in our NSCs	in other ChIP-seq (10)	in our NSCs	in other ChIP-seq (10)				%	n°	n°	
**Nkx2.1**	DA504	10,000	-	✓	-	-	✓	✓	+	94.7%	36/38	37/38	++++
**Sp8**	PA545	10,000	-	-	✓	✓	-	-	+	75.0%	15/20	15/20	+
	DA545		✓	✓	-	-	-	-	+	94.9%	37/39 (1)	39/39	++++
**Coup-TF1**	DA2467	12,000	✓	-	✓	✓	-	-	+	35.0%	7/20	7/20	+
	DA602	27,000	✓	-	-	-	-	-	+	40.0%	8/20	8/20	+
**Ntng1**	DA1414	100,000	✓(2)	✓	✓(2)	-	-	-	+/-	2.6%	(1-3)/39 (3)	(5-7)/39 (4)	+
**Irx1**	DA597	450,000	✓	✓	-	✓	✓	(6)	+	49.1%	29/59	53/59 (5)	++
**Socs3**	DA463	32,000	✓	✓	✓	✓	✓	(6)	+	23.5%	8/34 (7)	16/34	+
**Chd7**	DA1439	40,000	✓	✓	-	-	-	-	+	13.6%	3/22	9/22	++
**Sox3**	DA2733	350,000	-	- (8)	-	-	✓	✓	+	100.0%	130/130	130/130	+++++
	DA2702	65,000	✓	✓	-	-	-	-	+	81.2%	13/16	15/16	+++
**Sox4**	DA2458	650,000	✓	✓	✓	✓	✓	✓	+	93.4%	114/122	119/122	+++
**Cxcr4**	DA62	180,000	✓	✓	-	-	✓	(6)	+	31.2%	30/96	56/96	++
**Zfp335**	DA1303	125,000	✓	-	-	✓	-	-	-	0%	0/56	4/56	-
	PA1303		-	✓	✓	-	-	-	-	0%	0/25	5/25	-
**c-Fos**	DA852 (e1)	40,000	✓	-	✓	✓	✓	✓	+	22.2%	12/54	54/54 (9)	+++
	DA854 (e2)	20,000	✓	✓	✓	-	✓	✓	+	9.6%	7/73	73/73 (9)	+

(1): 10/37 GFP+ also in midbrain-hindbrain boundary;
(2): the peak is not completely included;
(3): one embryo with clearly positive signal, two with weak signal;
(4): five embryos with clearly positive signal, two with weak signal;
(5): 18/53 in more posterior neural tube regions;
(6): not present in VISTA enhancer;
(7): 2 embryos with clearly positive signal, 6 with weak signal;
(8): presence of a SOX3 peak;
(9): all transgenics showed some expression in the trunk;
(10): Lodato *et al.*, 2013; Engelen *et al.*, 2011.
