# Supplementary material for: Mapping the Global Chromatin Connectivity Network for Sox2 Function in Neural Stem Cell Maintenance
Source: Cell Stem Cell. 2019 Mar 7;24(3):462–476.e6. doi: 10.1016/j.stem.2019.02.004 (PMC6506828; doi:10.1016/j.stem.2019.02.004)
Supplement: Methods S1. PCR Primers for Anchor Amplification, Related to STAR Methods [file mmc7.pdf]

Methods S1, PCR primers for anchor amplification

(related to STAR Methods, Zebrafish transgenesis)

Gene associated with proximal anchor	Cloned anchor	Forward primer (5'->3')	Reverse primer (5'->3')
**Nkx2.1**	DA504	TCCCGTTCCTAGTCTTTGATACTT	CGAGCAACAGGAGAGGAATAATTT
**Sp8**	PA545	GACATCCAGACCTCTGTTTTCC	TCTACCAGAGGTGGGATTCAAG
**Sp8**	DA545	GGGGAAGAGTTCCTAGCCATT	GTGGGAGCTCAATTCATCTAA
**Coup-TFI**	DA2467	GCTCCAGCGTCTACTGAGAAAT	AGCAGAATCCCTGAGACTTCAC
**Coup-TFI**	DA231	CCAGTGAAACACCTACTCACCA	AAGTTGGCATTTTTAGGACTCG
**Ntng1**	DA1414	GTAGAGGCGCGGAACCATAG	GGGGTAAAAGGAAAGGGCAAA
**Irx1**	DA597	CAGCAAAGCATTGTAACTGTGA	TGGGGCTTTAACACAAGCAT
**Socs3**	DA463	GCTCACACTGACCCATAGGTTT	TTGCCTCTCAGAGTGAACCA
**Chd7**	DA1439	AGGCAAGCTCACCAGCTCT	GATTTCAAAGGCAGCCACAT
**Sox3**	DA2733	GGAGGCACATGAAAGCAATAA	GGGTAAGGTTAAAATGGCTTTTG
**Sox3**	DA2702	ACTGTCCATTTAGTTTTCATAAATCA	GTGGGCAGGGATACCTTAGTCT
**Sox4**	DA2458	GTCCTTCAGCAAGCTCTAAACA	AATGGTGGTGAAATCTGCAAGT
**Cxcr4**	DA62	GGACCCCTCAGTGAATATTAAGG	TTTGCACTGTGGTACACATTTT
**Zfp355**	DA1303	CTAGCACTCAACCCTGAGATT	GACTTCAGAATGGAGCCAGAAC
**Zfp355**	PA1303	AAGTAGTTCCGGTTCGAG	TTTGAGGCTTTCACTCTGCTG
**Fos**	DA852	CCAGGCCAGTCTTTTCACAC	TCGAGGCTAGTTTGGGTTGT
**Fos**	DA854	TAATCTTCTGACTCCCCGGC	GAGCTGGTGAGTGCAGTCTA
