# Supplementary material for: Mapping the Global Chromatin Connectivity Network for Sox2 Function in Neural Stem Cell Maintenance
Source: Cell Stem Cell. 2019 Mar 7;24(3):462–476.e6. doi: 10.1016/j.stem.2019.02.004 (PMC6506828; doi:10.1016/j.stem.2019.02.004)
Supplement: Document S2. Article plus Supplemental Information [file mmc8.pdf]

Cell Stem Cell

Article

Mapping the Global Chromatin Connectivity Network for Sox2 Function in Neural Stem Cell Maintenance

Graphical Abstract

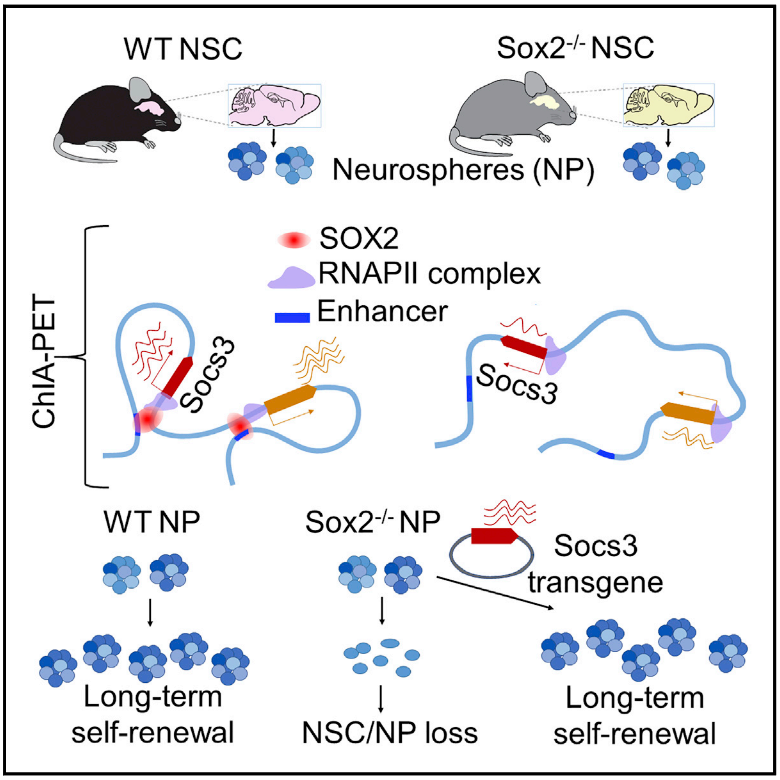

Authors

Jessica A. Bertolini, Rebecca Favaro, Yanfen Zhu, ..., Francois Guillemot, Silvia K. Nicolis, Chia-Lin Wei

Correspondence

silvia.nicolis@unimib.it (S.K.N.), chia-lin.wei@jax.org (C.-L.W.)

In Brief

Bertolini et al. report that long-range chromatin interactions in neural stem cells (NSCs) are enriched in Sox2-bound enhancers; in Sox2-deleted NSCs, interactions are reduced. Genes downregulated in Sox2-deleted cells are enriched in interactions with enhancers normally Sox2-bound. Overexpression of Socs3, a gene downregulated in mutant NSCs, rescues long-term NSC self-renewal.

Highlights

- Sox2-bound enhancers are enriched within long-range interactions in neural stem cells
- SOX2 loss decreases chromatin interactivity genome-wide
- Sox2-bound enhancers from interactions activate reporter genes in zebrafish forebrain
- Socs3, a gene downregulated in Sox2 mutant NSCs, rescues their self-renewal

Bertolini et al., 2019, Cell Stem Cell *24*, 462–476
March 7, 2019 © 2019 Elsevier Inc.
https://doi.org/10.1016/j.stem.2019.02.004

CellPress

Cell Stem Cell
Article

Mapping the Global Chromatin Connectivity Network for Sox2 Function in Neural Stem Cell Maintenance

Jessica A. Bertolini,[1,9] Rebecca Favaro,[1,9] Yanfen Zhu,[2,9] Miriam Pagin,[1] Chew Yee Ngan,[2] Chee Hong Wong,[2] Harianto Tjong,[2] Marit W. Vermunt,[3,8] Ben Martynoga,[4] Cristiana Barone,[1] Jessica Mariani,[1] Marcos Julián Cardozo,[5] Noemi Tabanera,[5] Federico Zambelli,[6] Sara Mercurio,[1] Sergio Ottolenghi,[1] Paul Robson,[2,7] Menno P. Creyghton,[3] Paola Bovolenta,[5] Giulio Pavesi,[6] Francois Guillemot,[4] Silvia K. Nicolis,[1,10,*] and Chia-Lin Wei[2,*]

[1]Department of Biotechnology and Biosciences, University Milano-Bicocca, 20126 Milano, Italy
[2]The Jackson Laboratory for Genomic Medicine, Farmington, CT, USA
[3]Hubrecht Institute-KNAW and University Medical Center Utrecht 3584CT, Utrecht, the Netherlands
[4]The Francis Crick Institute, Midland Road, London NW 1AT, UK
[5]Centro de Biología Molecular Severo Ochoa, Consejo Superior de Investigaciones Científicas-Universidad Autónoma de Madrid and Ciber de Enfermedades Raras (CIBERER), ISCIII Madrid, Spain
[6]Department of Biosciences, University of Milano, 20133 Milano, Italy
[7]Stem Cell and Regenerative Biology, Genome Institute of Singapore, Singapore
[8]Present address: Division of Hematology, The Children's Hospital of Philadelphia, Philadelphia, PA, USA
[9]These authors contributed equally
[10]Lead Contact
*Correspondence: silvia.nicolis@unimib.it (S.K.N.), chia-lin.wei@jax.org (C.-L.W.)

https://doi.org/10.1016/j.stem.2019.02.004

SUMMARY

The SOX2 transcription factor is critical for neural stem cell (NSC) maintenance and brain development. Through chromatin immunoprecipitation (ChIP) and chromatin interaction analysis (ChIA-PET), we determined genome-wide SOX2-bound regions and Pol II-mediated long-range chromatin interactions in brain-derived NSCs. SOX2-bound DNA was highly enriched in distal chromatin regions interacting with promoters and carrying epigenetic enhancer marks. *Sox2* deletion caused widespread reduction of Pol II-mediated long-range interactions and decreased gene expression. Genes showing reduced expression in *Sox2*-deleted cells were significantly enriched in interactions between promoters and SOX2-bound distal enhancers. Expression of one such gene, *Suppressor of Cytokine Signaling 3* (*Socs3*), rescued the self-renewal defect of *Sox2*-ablated NSCs. Our work identifies SOX2 as a major regulator of gene expression through connections to the enhancer network in NSCs. Through the definition of such a connectivity network, our study shows the way to the identification of genes and enhancers involved in NSC maintenance and neurodevelopmental disorders.

INTRODUCTION

Neural stem cells (NSCs) are critical for brain development and for postnatal maintenance of neurogenesis in specific brain areas. SOX2, a transcription factor (TF) essential for pluripotency (Avilion et al., 2003; Takahashi and Yamanaka, 2006), is also required for correct brain development. In humans, *SOX2* mutations cause genetically dominant nervous system disease involving hippocampus and eye defects, epilepsy, and learning disabilities (OMIM 206900). In mice, *Sox2* ablation causes similar defects, such as hippocampal hypoplasia, microcephaly, ventral forebrain depletion, and anophthalmia, some of which may result from a defect in NSC self-renewal (Favaro et al., 2009; Ferri et al., 2013). These *in vivo* defects are reflected in the inability of *Sox2*-deleted NSCs to self-renew in long-term cultures (Favaro et al., 2009). SOX2 functions and targets are the subject of intense investigation (Engelen et al., 2011; Gonçalves et al., 2016; Hagey et al., 2016; Lodato et al., 2013; Zhu et al., 2013).

Transcriptional regulation is mediated via DNA looping between gene promoters and their corresponding distal enhancers, often located at large distances and skipping intervening genes (de Laat and Duboule, 2013; Rivera and Ren, 2013; Sanyal et al., 2012; Zhang et al., 2013). Genome-wide analyses of long range interactions in chromatin (Sanyal et al., 2012; Zhang et al., 2013) define complex three-dimensional networks (the connectome), whereby a promoter may interact not only with enhancers but also with additional promoters, which are in turn connected to further promoter(s) and/or enhancer(s). The genome-wide connectome is cell-type specific (Gorkin et al., 2014; Zhang et al., 2013), presumably reflecting cell-type-specific transcription factor representation. So far, it is unknown to what extent a single transcription factor influences the function of genome-wide interaction networks in controlling cell-specific transcriptional activity.

We previously used chromatin interaction analysis with paired-end tag sequencing (ChIA-PET) to identify RNA polymerase II (Pol II)-mediated long-range interactions in embryonic stem cells (ESCs) and in brain-derived NSC or progenitor cell cultures (Zhang et al., 2013). In the present work, we sought to identify molecular mechanisms underlying *Sox2*-dependent gene regulation in NSCs, as well as genes involved in *Sox2*-dependent maintenance of long-term NSC self-renewal. We thus deleted *Sox2* in NSCs in mouse embryonic brain and

 © 2019 Elsevier Inc.

Table 1. Summary of the ChIA-PET Sequencing and Interaction Analysis

		wTR1	wTR2	wTR3	mTR1	mTR2	mTR3
1	Total PETs with linker	143.3 M	35,295,370	33,767,613	151.9 M	24,976,862	21,514,824
2	Mapped PETs	143.1 M	23,672,163	20,624,486	151.7 M	16,512,951	11,050,816
3	Unique PETs	6.8 M	22,208,305	16,873,008	22.6 M	15,756,748	10,160,601
4	Self-ligated PETs	684 K	4,296,406	2,869,465	2.7 M	2,185,670	1,729,424
5	Number of Pol II binding sites ($p < 1e-5$)	11,819[a]	41,187[a]	36,641[a]	12,068[a]	21,020[a]	36,139[a]
6	Intra-molecular chromatin ligated PETs	691 K	13,105,813	10,207,155	1.3 M	9,360,576	6,119,151
7	Significant interactions (loops) (FDR <0.05, $p < 0.05$)	7,046[a]	96,295	63,458	2,984[a]	29,713	15,561
8	Significant loops with Pol II peaks on both anchors		18,022[a]	7,346[a]		2,878[a]	3,202[a]
9	Number of significant loops per million intra-chr PETs	10,197	7,348	6,217	2,295	3,174	2,543
10	Number of significant Pol II-bound loops per million intra-chr PETs		1,375	720		307	523

ChIA-PET data in triplicates were processed to define the binding peaks and significant interactions (see STAR Methods). For full list of significant interactions, see Table S1. PET, paired end tag; unique PETs, PETs for which the sequence of either side of the linker (e.g., the biotinylated linker in Figure S1B) can be uniquely mapped to one specific point in the reference genome.
[a]Numbers of the significant interactions used in further analyses are indicated.

studied the effects of embryonic loss of *Sox2* on RNA expression in neonatal NSCs grown *in vitro* (see Favaro et al. 2009) and its relationship to the Pol II-mediated chromatin long-range interaction network. We identified thousands of genes connected via long-range interactions to distal SOX2-bound, epigenetically defined enhancers; many of these genes, including important neurodevelopmental genes, were downregulated upon *Sox2* ablation. We validated one of these as a critical downstream SOX2 target whose re-expression in *Sox2* mutant NSCs is sufficient to rescue their self-renewal defect.

RESULTS

Comparison of Genome-wide Pol II-Mediated Long-Range Chromatin Interactions in Wild-Type and Sox2-Deleted NSC

We established NSC cultures from the neonatal forebrain of conditionally (at E11.5) *Sox2*-ablated mice and their control non-deleted littermates (Favaro et al., 2009). Freshly isolated *Sox2*-deleted (mutant; MUT) and control (wild-type; WT) NSCs efficiently expand in culture at early passages (Favaro et al., 2009), however, MUT NSCs later fail to self-renew long-term, pointing to a requirement for *Sox2* in NSC maintenance that matches a defect observed also *in vivo* after P0 in the hippocampus (Favaro et al., 2009). Sox2-deleted NSCs retain the ability to differentiate into glia and neurons upon differentiation induction, however, under self-renewal culture conditions, they do not spontaneously differentiate, as indicated by morphological and immunochemical criteria and by comparison of RNA sequencing (RNA-seq) data (by Pearson correlation, hierarchical clustering, and principal component analysis) of un-induced WT and MUT cells (day 0) with WT cells induced to initial differentiation (day 4) (data not shown).

To determine the effect of *Sox2* loss on the genome-wide pattern of Pol II-mediated long-range chromatin interactions, we first performed ChIA-PET analysis with anti-polII antibodies, specific for the preinitiation complex (Zhang et al., 2013), comparing *ex vivo* NSC cultures derived at P0. These cultures express forebrain-specific transcripts, indicating that the NSCs maintain a forebrain identity (Zappone et al., 2000; Zhang et al., 2013). ChIA-PET identifies protein-mediated genome-wide long-range chromatin contacts through proximity ligation and chromatin immunoprecipitation (ChIP). We generated ChIA-PET data from both normal (WT) and *Sox2*-ablated (MUT) brain cells to determine Pol II-mediated long-range connectivity (distant enhancer-promoter and promoter-promoter connectivity) (Figure S1). We began to analyze the chromatin connectivity by following the original version of the protocol (Zhang et al., 2013) that performed nuclei lysis followed by proximity ligation and Pol II ChIP from hundreds of million pooled NSCs from WT and Sox2-deleted (MUT) neonatal (P0) forebrains (4 WT and 6 MUT, deleted at E11.5) (Favaro et al., 2009) neonates. These datasets (Table S1) are referred to as wTR1 and mTR1, respectively. We observed a substantial reduction of normalized interactions (numbers of significant interactions per million intra-molecular ligation PETs) in *Sox2*-deleted (2,295 in mTR1) versus WT NSCs (10,197 in wTR1) (Table 1 and Figure 1, discussed below). We further verified that such reduction of overall Pol II connectivity observed in MUT NSCs did not result from different Pol II immunoprecipitation efficiencies between WT and MUT cells; indeed, we observed highly similar normalized density profiles between WT and MUT genomic regions (Figure S2). Specifically, the vast majority (92%) of the ChIA-PET-defined Pol II binding regions in WT NSCs were retained in MUT NSCs, irrespective of whether or not they were connected (Figures 1 and S2). To confirm that the reduction of Pol II-mediated interactions in MUT NSCs was not influenced by the pooling of heterogeneous samples or correlated with the experimental procedure, we subsequently generated additional replicate datasets using an improved ChIA-PET method (named *in situ* ChIA-PET) (Figure S1). In the *in situ* Pol II ChIA-PET protocol, instead of performing proximity ligation in chromatin mixtures from hundreds of million cells that is intrinsically noisy (as evident by the high level of inter-chromosomal PETs), proximity ligation was performed in permeabilized intact nuclei, followed by ChIP and a transposase-mediated “tagmentation” to generate PETs for sequencing analysis (Figure S1; STAR Methods). As such, the *in situ* ChIA-PET method results in higher efficiency in capturing

CellPress

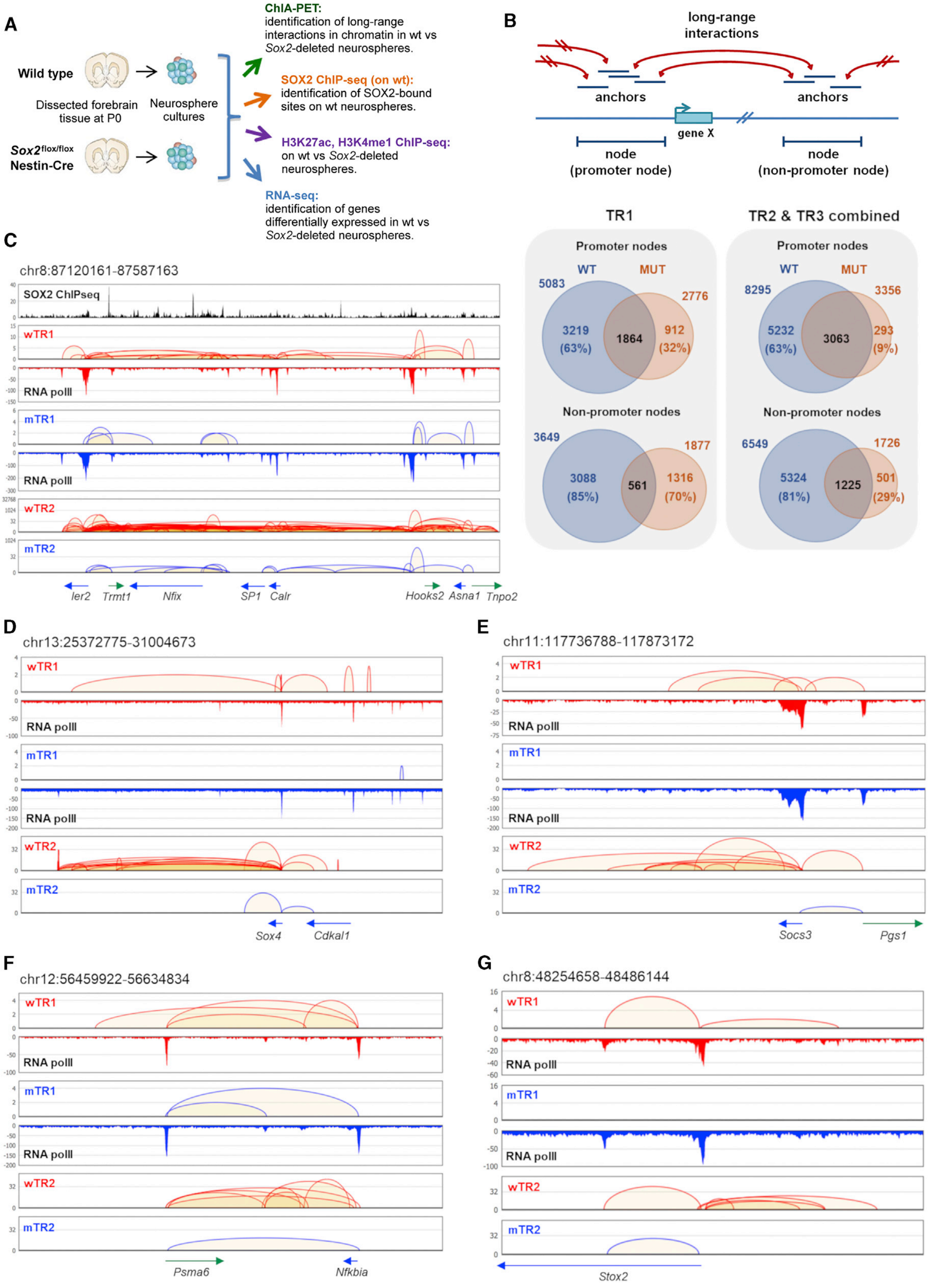

(legend on next page)

intra-molecular ligation PETs, thus requires significantly lower numbers of cells to yield highly sensitive detection of protein-mediated chromatin interactions (Mumbach et al., 2016; Table 1). Therefore, this approach allowed us to analyze cultures from individual neonatal forebrains (two WT, wTR2 and wTR3, and two MUTs, mTR2 and mTR3). The improvement can be shown by the ratio of the intra-molecular interaction PETs; in the TR1 (original version), only 5%–10% of the uniquely aligned paired reads (unique PETs, Table 1, line 3) were intra-molecular chromatin ligated PETs (defined by *cis*-interaction PETs, Table 1, line 6), whereas in TR2 and TR3, 50%–60% of the uniquely aligned paired reads were intra-molecular chromatin ligated PETs (Table 1). Despite two versions of the protocols being applied, the three ChIA-PET experiments (TR1, TR2, and TR3) showed overall highly similar interaction patterns when the same genotypes (WT or MUT) were considered, at different resolution (Figure S2); 74% and 80% of the interactions detected in wTR1were also detected in the combined (i.e., the sum of) wTR2 and wTR3 interactions (within a window ± 5 kb and ± 10 kb, respectively). Further, the average reproducibility score between wTR2 and wTR3 based on SCC (stratum-adjusted correlation coefficient) (Yang et al., 2017) over all 20 chromosomes was 0.935 and 0.839 for wTR2-wTR3 and mTR2-mTR3, respectively (Table S2).

The *in situ* proximity ligation adopted in the improved ChIA-PET method effectively captures specific Pol II-mediated long-range interactions between regulatory elements through the Pol II ChIP enrichment (Figure S1). From a total of 6–13 million intra-molecular PETs in WT and MUT TR2 and TR3, we defined between 15,000 to 96,000 "significant interactions (loops)" (false discovery rate [FDR] <0.05, $p < 0.05$), enriched in Pol II-mediated interactions) (Table 1, line 7; Figure S1; STAR Methods). Among these significant interactions from TR2 and TR3, 2,878–18,022 interactions were mediated by Pol II (defined by the interactions with Pol II binding at *both* DNA regions connected by the interactions) (Table 1, line 8, and STAR Methods). We defined DNA regions connected by an interaction as "anchors" and overlapping anchors as "nodes" (Figure 1B). We further annotated nodes and anchors as promoter nodes or anchors if they resided within 2.5 kb from annotated transcription start sites (TSS) and the remaining ones as non-promoter nodes/anchors. Based on PhastCons Score Threshold analysis, these non-promoter nodes were significantly more conserved among vertebrate genomes relative to random intergenic regions, suggesting their potential function in chromatin organization (not shown).

Similarly to mTR1 NSCs, mTR2 and mTR3 exhibited a global reduction in the numbers of chromatin interactions from both the general chromatin contacts and the specific Pol II-mediated interactions if compared to WT NSCs (wTR2 and wTR3, respectively) (Figure 1B). The number of normalized significant interactions ("number of loops detected per million of intra-molecular ligated reads") ranged between 6–10 K in WT but were reduced to only 2–3 K in MUT cell samples (Table 1, line 9), while the normalized significant Pol II-mediated interactions were 719 and 1,374 in WT, but only 307 and 525 in MUT cells samples, respectively (Table 1, line 10). Consistent with the reduction of the Pol II-mediated interactions in MUT cells, the number of nodes was lower in MUT cells (Figure 1B). Indeed, from 8,295 promoter nodes and 6,549 non-promoter nodes in combined wTR2 and wTR3, the corresponding numbers in MUT cells were reduced to 3,356 and 1,726, respectively. On the other hand, the majority of the nodes observed in mTR2 and mTR3 samples were detected in WT cells as well. These changes of interactions can be found in many specific loci. The changes were not uniform across the genome, but rather highly variable, exhibiting loci showing drastic reduction interspersed with loci showing little or no reduction (Figure 1; see also Figures S3 and S5, Table S3, and screenshots throughout the paper). In some cases, while the data from mTR1 showed apparent loss of interactions (as compared to wTR1), the data obtained by the more sensitive *in situ* ChIA-PET, providing a higher depth in the intra-molecular ligated PETs, showed a reduction in frequency instead of a complete loss of the interactions (Figures 1D and 1G, compare TR2 with TR1; see also Figure S3); the above discussed dependence of the detection of some interactions on obtaining high numbers of interacting PETs makes it difficult to prove the complete loss of any specific interaction. In some regions, we actually observed new sets of loops emerging in MUT cells (Figure 1).

In conclusion, the data indicate that in the absence of *Sox2*, chromatin connectivity was substantially altered genome-wide, with an overall decreased interaction frequency, in particular at selected loci.

SOX2-Bound Distal Regions Carrying Enhancer Marks Are Highly Enriched Within Interactions

The changes in connectivity observed following *Sox2* ablation point to a role for SOX2 DNA binding within chromatin in the generation and/or maintenance of long-range interactions. We thus identified SOX2-bound sites through genome-wide ChIP-seq of WT brain-derived NSCs in culture (Figure S4; STAR Methods). We also performed ChIP-seq, in both WT and MUT NSCs, for histone modifications H3K27ac and H3K4me1 (Figure S4, two replicates), allowing for the identification of active ($H3K27ac^{+}$

Figure 1. *Sox2* Ablation Causes Major Loss of Long-Range Interactions in Brain-Derived NSCs
(A) Functional genomics analyses.
(B) Top: "anchors" and "nodes" connected by long-range interactions; bottom: numbers of promoter/non-promoter nodes in WT and MUT NSCs, left: TR1 and right: TR2 and TR3 combined.
(C–G) Connectivity diagrams in WT NSCs (WT interactions; red) and MUT NSCs (MUT interactions; blue), across 5 different chromosome regions, in the wTR1, wTR2, and mTR1, mTR2 analysis; regions coordinates are: chr8:87120161-87587163 (C), chr13:25372775-31004673 (D), chr11:117736788-117873172 (E), chr12:56459922-56634834 (F) and chr8:48254658-48486144 (G). Their genomic coordinates are indicated above each panel, and genes within each region shown below the panels. Pol II- and SOX2-binding peaks are shown. PET counts (Y axes); note different (log10) scales in some panels. In MUT NSCs, an overall decrease of "looping" is seen, but some interactions are lost, others are maintained. Note the persistence of Pol II binding in MUT NSCs and the frequent coincidence (in C) of SOX2 peaks with interaction anchors.
See also Figures S1, S2, S3 and Table 1.

and H3K4me1^{+}), as well as "poised" (H3K4me1^{+} only) enhancers, with the potential to be activated (Cantone and Fisher, 2011; Creyghton et al., 2010; Rivera and Ren, 2013). For the latter analysis, we used both a "peak-calling" and a "segmentation" approach (chromHMM), which both led to qualitatively consistent results (see STAR Methods).

Finally, we linked SOX2-binding sites to both epigenetic marks and interacting anchors, as defined in WT NSCs by Zhang et al. (2013) (corresponding to wTR1) and in *in situ* ChIA-PET experiments (wTR2, wTR3) (Figure 2). For a summary of data, see Tables S4 and S5.

SOX2-bound sites were rarely located at promoters (±1 kb TSS of RefSeq genes) and more frequently in intronic and intergenic distal regions (Figure 2A and data not shown). Over 90% of SOX2-bound sites were associated with nucleosomes characterized by the presence of either or both the histone modifications investigated in WT NSCs (Figure 2A, histograms: peak calling and chromHMM); in particular, both SOX2-bound promoters and distal regions were mostly (90%) H3K27ac^{+}. On the other hand, within H3K27ac^{+} regions, a much smaller proportion of promoters than of distal regions were SOX2-bound (Figures 2B, right, and S4); indeed, within distal H3K27ac^{+} regions, SOX2-bound regions were highly enriched in comparison to 1,000 sets of random genomic loci ($p < 0.001$, random sampling, see STAR Methods).

To identify the binding of SOX2, if any, within long-range interactions, we first classified interactions in WT cells according to the type of interacting element (i.e., promoter [P] or non-promoter [non-P]) (Figure 2C). Approximately 85%–90% of the interactions were mediated through promoters (promoter-promoter [P-P] or promoter-non-promoter [P-non-P] interactions) in both WT and MUT cells, equally subdivided between P-P and P-non-P classes (Figure 2C); within P-non-P interactions, a promoter is connected to either an intergenic or an intragenic region. Only a small number (10%–15%) of interactions connect two non-promoter regions (Figure 2C). An almost identical distribution was observed using interactions defined from TR2 and TR3 (Figure 2C).

Within interactions, SOX2 peaks were highly abundant; ca. 35%–46% of all interactions in WT NSCs (TR1, TR2, and TR3) carried a SOX2-bound site within at least one of the two interacting anchors (Figure 2D; Tables S4 and S5). Specifically, approximately half (44%–53%) of P-nonP interactions (also called P-E, see below) were SOX2-positive with 34%–43% of distal elements (putative enhancers) being SOX2-bound (Figure 2D). Approximately 95% of SOX2-bound distal anchors were in regions carrying both active enhancer marks (H3K27ac^{+}, H3K4me1^{+}) and the remainder with either one or the other mark; among non-SOX2-bound distal anchors, ~70% were associated with both marks and ~15% with either (not shown). From now on, we refer to these interactions as "promoter-enhancer" (P-E) interactions. Importantly, SOX2-positive epigenetically marked (EM) distal regions (whether H3K27Ac^{+} and/or H3K4Me1^{+}) were significantly more involved in interactions than SOX2-negative EM regions: this was observed in the original ChIA-PET (TR1), as well as in *in situ* ChIA-PET data (TR2 and TR3) (Figures 2E and S4 and data not shown). Thus, the high frequency of SOX2-bound distal anchors is the result of both the enrichment of SOX2 binding within EM distal regions (Figure 2B) and the preferential engagement in long-range interactions of SOX2-positive EM regions versus non-SOX2-bound regions (Figure 2E).

In conclusion, the high enrichment of SOX2-positive EM regions within distal anchors in P-E interactions points to a functional role of SOX2 binding at the level of these interactions.

Interestingly, the loss of SOX2 in MUT NSCs does not lead to important changes (loss or gain) in the patterns of histone modifications: enrichment in H3K27ac, H3K4me1, both, or none (Figure S4).

SOX2-Dependent Long-Range Interactions Predict Novel Forebrain Enhancers

The strong enrichment of anchors in epigenetic EMs suggested that long-range interaction anchors might be used to identify regulatory elements driving gene expression in the developing brain (Figures 3 and S5; Table S6). We identified several genes playing important developmental roles in the forebrain, some of which are homologs of human genes involved, when mutated, in inherited brain diseases (microcephaly, intellectual disability, etc.) (Table S6), which were connected by multiple P-E (and P-P) interactions to SOX2-bound elements (possible enhancers). Some of the distal anchors connected to these genes by P-E interactions overlapped with previously identified p300-bound enhancers, already shown to be active in forebrain as transgenic constructs (VISTA enhancers, https://enhancer.lbl.gov) (Visel et al., 2009; Figures 3 and S5). Interestingly, VISTA enhancers were enriched within TR1, TR2, and TR3 interaction anchors (with 13% to 23% of VISTA enhancers overlapping with anchors), and particularly so within distal non-promoter interaction anchors; in *Sox2* MUT cells the proportion of VISTA enhancers overlapping with anchors dropped to ca. 4%, and their representation in distal anchors was drastically reduced (Figure S5). These data point to potential functional roles of distal non-promoter anchors identified by ChIA-PET in gene regulation *in vivo* in the developing brain.

Next, we tested distal regions involved in SOX2-bound long-range interactions using a transgenic enhancer assay in zebrafish. Fifteen out of seventeen reporter constructs containing SOX2-bound distal anchors directed GFP expression to the developing forebrain (Figure 4; Table S7). GFP expression matched part of, or the whole, forebrain expression pattern of the endogenous zebrafish ortholog of the mouse gene connected to the analyzed enhancer (Figure 4). Similar data were obtained with anchors connected to important regulators of forebrain development, including *Sp8*, *Cxcr4*, *Sox3*, *Nr2f1*, *Irx1*, *Socs3* and *c-fos* (Figure 4; Table S6). Further, the expression of 3 out of 7 enhancers tested was affected by anti-*Sox2* morpholinos or by injected *Sox2* mRNA (Figure S6 and data not shown). Thus, RNA-polII-mediated, SOX2-bound interactions identify with high confidence novel forebrain enhancers.

SOX2-Bound Promoter-to-Enhancer Long-Range Interactions Are the Main Determinant of SOX2-Dependent Transcription

To correlate the expression of genes to the observed pattern of long-range interactions, we analyzed by RNA-seq the transcriptomes of WT and MUT NSCs (Tables S4 and S5).

To determine how the presence of Pol II-mediated interactions is reflected into gene expression levels, we subdivided genes

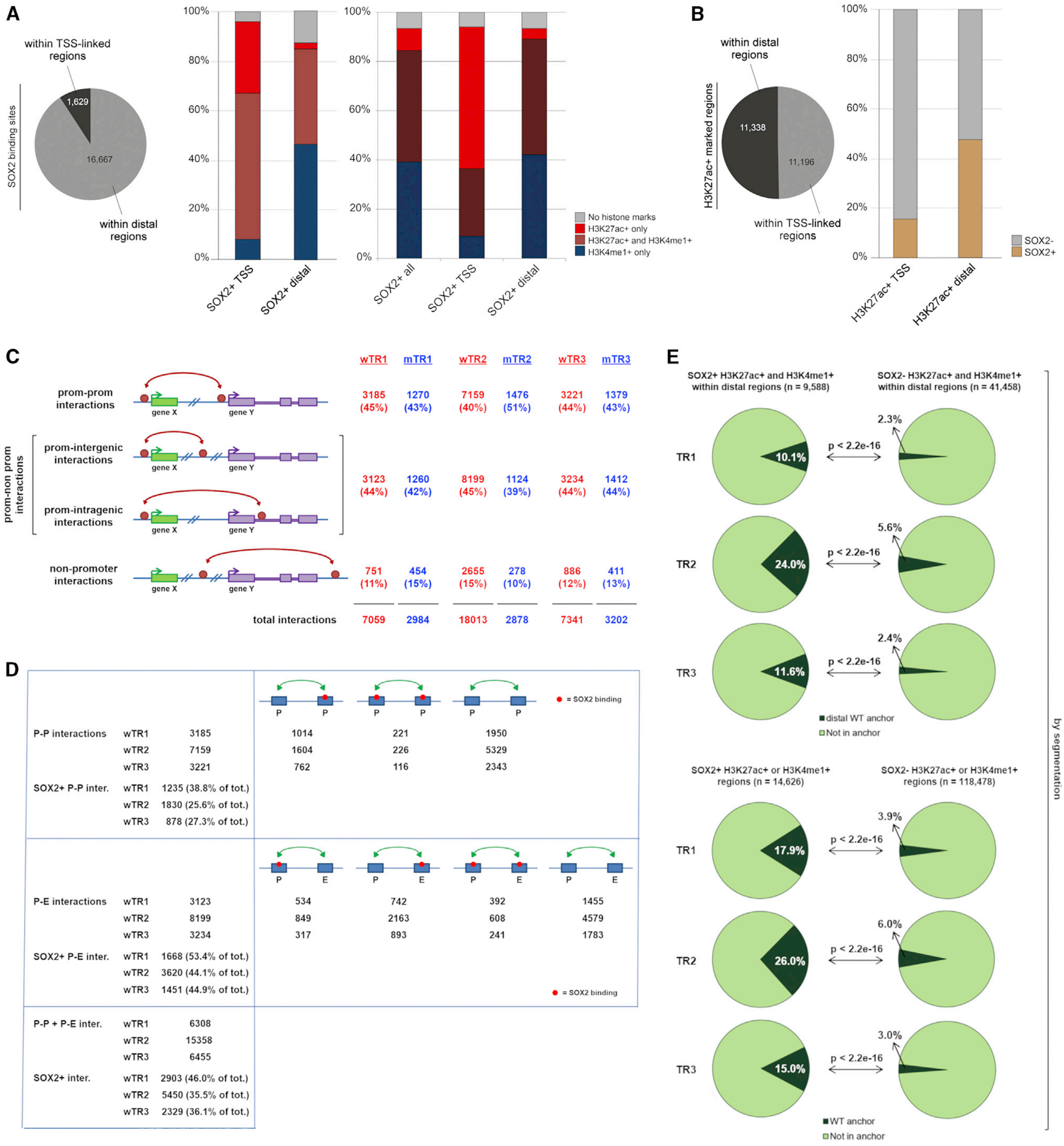

Figure 2. SOX2-Bound Regions Carrying Epigenetic Enhancer Marks (EM) Show Significantly Higher Overlap with Anchors Than SOX2-Negative EM-Positive Regions

(A) Left: number of SOX2-bound sites in regions linked to annotated TSS (±1,000 nt) and in distal, non-P regions. Right (histograms): percentage of different enhancer marks (EMs)-positive regions within SOX2-bound TSS-linked (SOX2$^+$ TSS) or distal (SOX2$^+$ distal) regions (left histograms, peak calling; right histograms, chromHMM).

(B) Fraction of SOX2-bound sites within EM-positive regions (H3K27Ac$^+$) on TSS-linked or distal regions (peak calling).

(C) Interaction types according to the nature of the connected regions, for wTR1, wTR2, wTR3, mTR1, mTR2, and mTR3. "Prom," annotated TSS-containing region (i.e., promoter).

(D) Numbers of P-P and P-nonP (P-E) SOX2-positive interactions in WT cells in wTR1, wTR2, and wTR3. See also Table S4.

(E) Fraction of SOX2$^+$ (left) versus SOX2$^-$ (right) EM-positive regions that overlap with anchors in wTR1, wTR2, and wTR3. Top: distal epigenetically marked regions (H3K27Ac$^+$ and H3K4me1$^+$) overlap with distal anchors. Bottom: all epigenetically marked regions (H3K27Ac$^+$ or H3K4me1$^+$) overlap with all anchor types, chromHMM. See also Figure S4 and Tables S4 and S5.

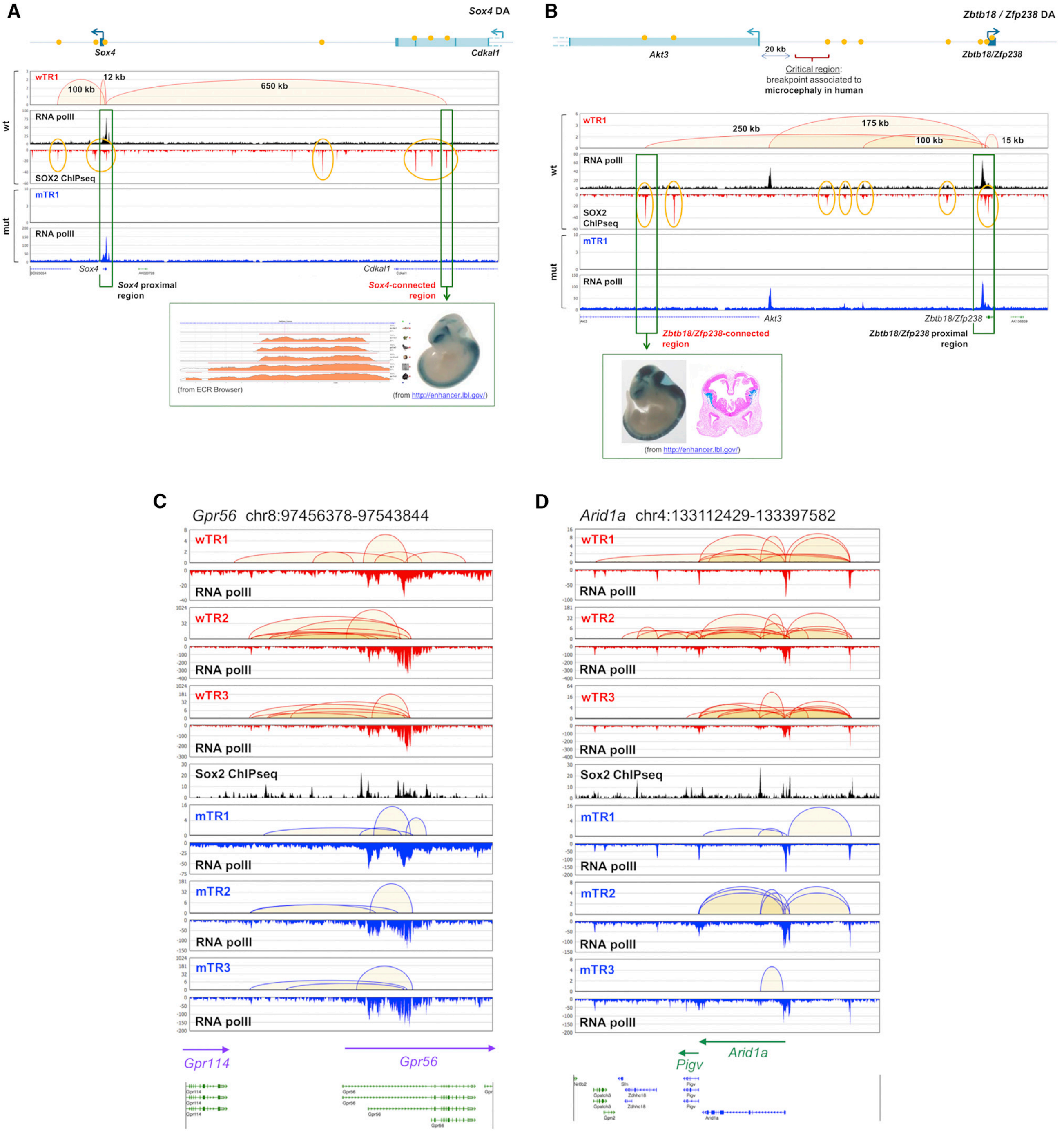

Figure 3. Distal Anchors Connected by *Sox2*-Dependent Interactions to Genes Important in Neural Development and Disease

(A and B) *Sox2*-dependent ChIA-PET interactions (TR1) between two different genes (*Sox4*, A; *Zbtb18*, B) and distal regions overlapping previously characterized "VISTA" enhancers (Visel et al., 2009); SOX2 ChIP-seq peaks (present paper), lacZ-stained transgenic embryos (from https://enhancer.lbl.gov), and evolutionary conservation (ECR browser).

(C and D) *Sox2*-dependent interactions (wTR1, wTR2, wTR3, mTR1, mTR2, and mTR3) involving *Gpr56* (C) and *Arid1a* (D), two genes whose human homologs are involved in neurodevelopmental brain disease.

See also Figure S5 and Tables S5 and S6.

according to the Pol II ChIA-PET connectivity of their promoters (Zhang et al., 2013) (non-connected promoter, promoter connected with promoters or enhancers, or promoter connected specifically with enhancers, see Figure 2D). Then, we determined the distribution of transcript levels in both WT and MUT NSCs for the different interaction categories (Figure 5A). Considering only

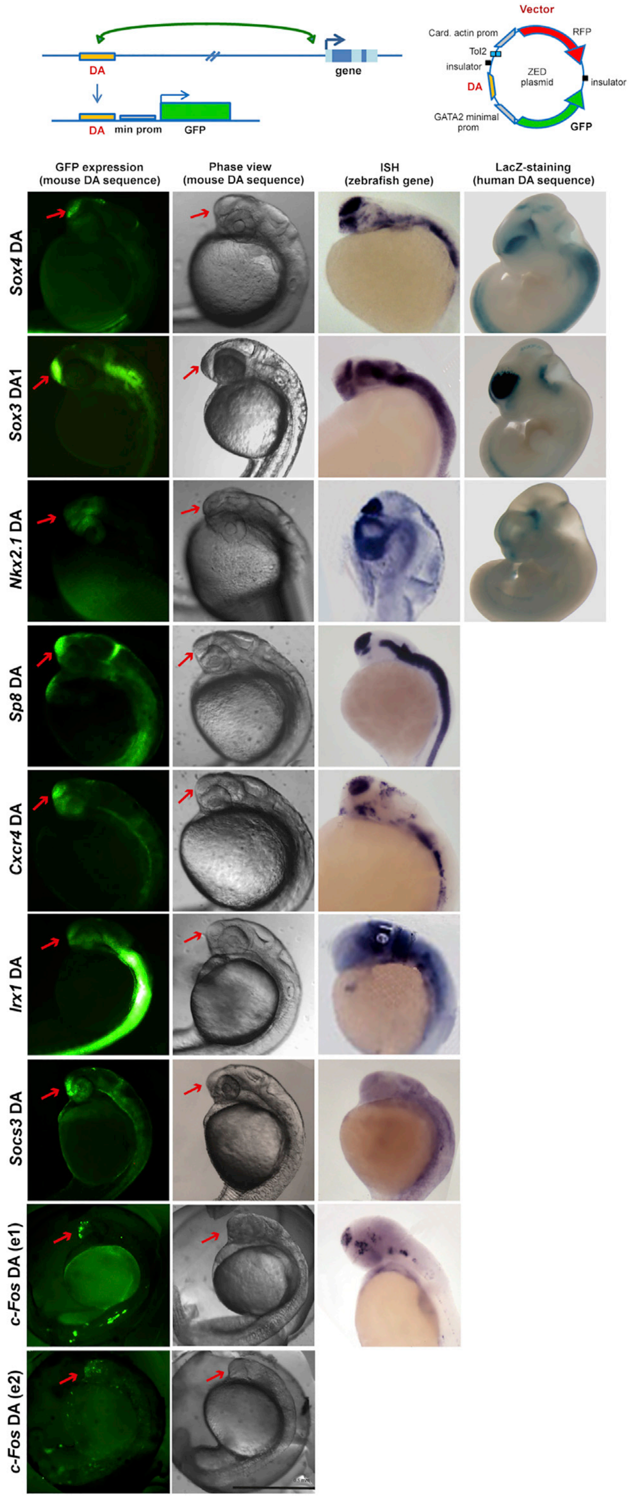

Figure 4. Distal Anchors in *Sox2*-Dependent Interactions Drive GFP Transgene Activity to Zebrafish Brain

Top: enhancer-dependent GFP-reporter (ZED): distal anchors (DA) from *Sox2*-dependent interactions are cloned upstream to a minimal promoter and GFP. Bottom: first and second left columns, GFP expression in transgenic embryos and bright-field images (F1 of stable lines, except for c-*fos*, transient transgenics). Third column: expression (*in situ* hybridization from http://zfin.org) of the endogenous zebrafish gene corresponding to the gene connected, in mouse, to the tested anchor. Fourth column: forebrain lacZ staining driven by transgenes carrying the human enhancers corresponding to the anchor (from https://enhancer.lbl.gov) (Visel et al., 2009).
See also Figure S6 and Table S6.

transcribed genes, those involved in interactions (P-P and P-E) showed a distribution of expression levels shifted toward higher values than the overall population of expressed genes (data for TR1, TR2, and TR3, Figure 5A); the highest expression levels were seen with genes involved specifically in interactions with enhancers (P-E) (Figure 5A) (p value $< 2.2 \times 10^{-16}$). Interestingly, higher expression levels were also associated with an increase in number of interactions per gene, particularly with P-E interactions, which show a clear trend toward higher expression values for every added "enhancer" (TR1, TR2, and TR3, Figure 5B). Moreover, genes whose promoter interacted with SOX2-bound enhancers were more expressed than those connected to enhancers not bound by SOX2 (TR1, TR2, and TR3, Figure 5A). Very similar results were obtained by both the analysis of data obtained by the original ChIA-PET (Zhang et al., 2013) (TR1) and by *in situ* ChIA-PET (TR2 and TR3).

We next compared gene expression between MUT and WT NSCs. In MUT NSCs, the distributions of expression levels of all genes, and of each category of genes, had lower median and mean values than in WT NSCs, with an overall distribution significantly shifted toward lower values (Figure 5A). Indeed, a Wilcoxon paired signed-rank test (see STAR Methods) showed that the differences between WT and MUT distributions of gene expression were highly significant (p value $< 2.2 \times 10^{-16}$ for every pair considered), for TR1, TR2, and TR3 (Figure 5A). To further assess the significance of the observed expression decrease in MUT NSC, we also plotted the distribution of the variation of the expression of each gene between WT and MUT cells, defined as log-fold ratios (log2 [TPM_wild-type/TPM_mutant]) (TPM, transcripts per million) (Figure 5C). To avoid bias from genes with low transcript levels, we considered only genes with TPM >1. The plot was clearly shifted toward positive values, indicating that the majority of genes were more highly expressed in WT than in MUT NSCs (Figure 5C).

Taken together, the above results indicate that loss of *Sox2* is associated with an overall gene expression decrease that is more relevant for genes involved in Pol II-mediated P-E interactions in WT NSCs; moreover, genes with SOX2-positive P-E interactions were more expressed than genes with P-E interactions in general. To further validate these results, we considered genes with TPM >5 in either WT or MUT cells and divided them into three groups (Figure 5D): group 1, genes showing a significant decrease of expression in MUT versus WT NSCs (677 genes); group 2, genes showing a visible but not statistically significant decrease of expression in MUT (2194 genes); group 3, all the other genes with TPM >5 (see STAR Methods). We then considered the different types of interactions associated with genes to determine whether genes in each expression variation group could be significantly associated with any type of interaction (i.e., if their number, within a given interaction class, was higher or lower than the number expected by chance). We summarized the results by defining a "co-association score" (see

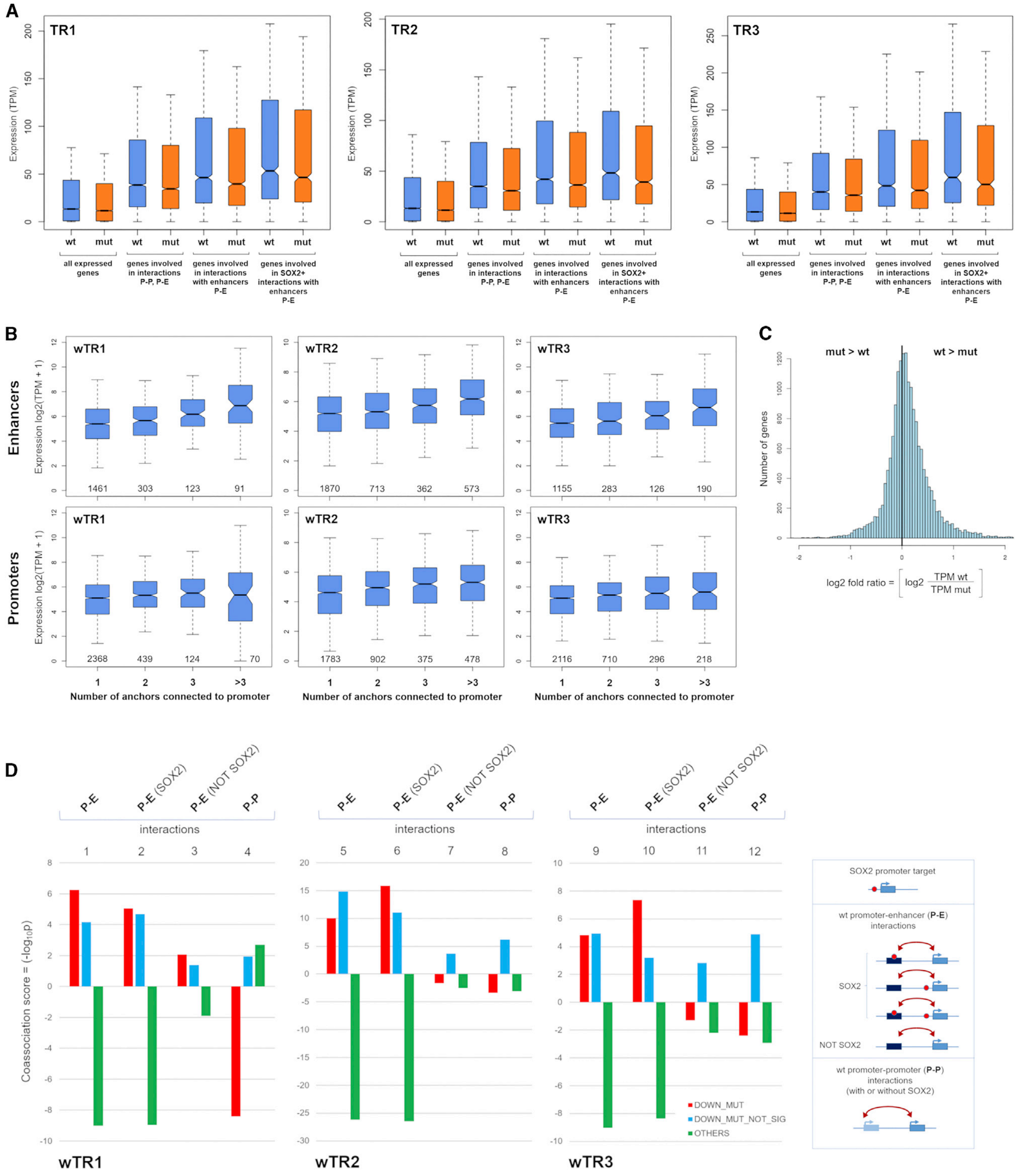

Figure 5. Reduced Gene Expression in *Sox2* MUT NSCs Correlates with Loss of Long-Range Interactions

(A) Distribution of expression values (TPM) of genes with TPM >0. Blue, WT NSCs; orange, MUT NSCs. From left to right: all genes; genes whose promoter is a node (P-P, P-E interactions); genes whose promoter is connected to an enhancer (P-E interactions); genes with SOX2-positive P-E interactions.

(B) Distribution of expression values (y axis) of genes according to the number and type of element (enhancer or promoter anchors) interacting with the gene promoter (x axis) in wTR1, wTR2, and wTR3. Top: interactions with enhancers. Bottom: interactions only with promoters. The number of genes involved is shown in each diagram inside the box along the x axis.

(C) Distribution of the fold ratio values for all genes with TPM >0 defined as log2 (TPM_WT/TPM_MUT). It confirms results shown in (B): the fold ratio is shifted toward positive values (i.e., a majority of genes have expression in WT higher than in MUT NSCs).

(legend continued on next page)

STAR Methods) as the -$\log_{10} p$ of the probability of observing, in each of the three expression groups, a given number of genes associated with a given type of interaction. We denoted a number lower than the expected value by multiplying the result by -1 (Figure 5D; Table S8). In all three experiments (wTR1, wTR2, and wTR3), the results confirmed a highly significant overlap between genes showing significantly decreased expression in MUT NSCs (group 1 genes) and those characterized, in WT NSCs, by a promoter to enhancer interaction (Figure 5D, lanes 1, 5, and 9). Moreover, the influence of SOX2 binding was evident by comparing group 1 genes connected to enhancers bound by SOX2, which yielded highly significant coassociation scores (lanes 2, 6, and 10), with those connected to enhancers not bound by SOX2 (lanes 3, 7, and 11), which yielded only marginally significant scores in wTR1 and nonsignificant scores in wTR2 and wTR3. On the other hand, SOX2 binding to promoters was only marginally associated to genes showing significantly decreased expression levels (p value >0.01, not shown), pointing to the fact that binding of SOX2 to a connected distal enhancer is much more functionally effective than its binding to a promoter. Finally, the P-P interactions category had no significant overlap with genes showing significant expression changes (group 1). However, group 2 genes showing only mildly decreased expression in MUT cells (not reaching the threshold requested for statistical significance), were significantly associated with the P-P interaction category (lanes 4, 8, and 12); we speculate that P-P interactions might be responsible for moderate positive effects on transcription, and their loss in MUT cells might predominantly result into a minor decrease of expression (as observed for group 2 genes), rather than into the stronger decrease observed with group 1 genes.

In conclusion, the identification of thousands of long-range interaction enhancers in NSCs, many of which are bound by SOX2, demonstrates an important role of SOX2 in controlling gene expression at the connected promoter.

Overexpression of *Socs3*, a Multi-Connected SOX2 Target, Rescues Long-Term Self-Renewal of MUT NSC

Sox2 MUT NSCs have a severe self-renewal defect, and their growth in culture becomes exhausted after 7–10 passages (~30 days) (Favaro et al., 2009). To evaluate if any specific gene (from among those whose expression is affected by *Sox2* loss) was able to rescue long-term self-renewal in MUT NSC, we expressed in MUT NSCs the *Socs3* gene, an inhibitor of Jak/Stat signaling, which antagonizes precocious differentiation of NSCs into astroglia (Cao et al., 2006). *Socs3* is strongly downregulated (down to 10%–15% of WT values) in MUT NSCs (Tables S4 and S5) and shows both a SOX2 peak on the promoter and multiple interactions (Figure 6A; see also Figure 1E), including one with a SOX2-bound anchor that already tested active in transgenic Zebrafish assays (Figure 4). We transduced both WT and *Sox2* MUT NSCs with a lentiviral *Socs3*-vector coexpressing GFP, performing three experiments at virus-to-cell ratios transducing 20%, 30%, or 50% of the NSCs. *Socs3*-transduced WT NSCs grew at a similar rate as untransduced WT NSCs and continued to grow long-term, whereas untransduced MUT NSCs stopped growing between passages 8–12 (Figures 6B and 6C). In contrast, *Socs-3* transduced MUT NSCs continued to grow long term, even after the untransduced MUT NSCs had stopped growing and could eventually be grown in bulk to generate large cell populations. At the time of initial divergence of the growth curves of transduced and untransduced MUT NSCs (experiment 3), most or all neurospheres (from transduced MUT NSCs) contained GFP^+ cells, and over 70% of the cells were positive by fluorescence-activated cell sorting (FACS), indicating a strong enrichment of the transduced cells; eventually, all cells became GFP^+ (Figure 6D). Note that *Socs3*-transduced WT NSCs were not positively selected relative to untransduced WT NSCs, indicating that in WT NSCs, the endogenous SOCS3 level was not limiting for optimal growth (not shown). This result identifies a SOX2-regulated gene, involved in SOX2-dependent interactions, whose abnormal regulation in MUT NSCs may be responsible for their defective long-term maintenance.

DISCUSSION

We show that SOX2 is critically involved in long-range chromatin interactions in NSCs; its ablation during early mouse development leads to a predominant decrease in long-range chromatin connectivity, particularly at some loci, when tested in neonatal forebrain-derived NSC cultures. SOX2 binding is greatly enriched on DNA regions connected by interactions (anchors) at either promoters or enhancers. The loss of *Sox2* decreases the expression of ~1,000 genes. The identification of thousands of epigenetically defined enhancers involved in long-range interactions allowed us to demonstrate that SOX2-bound long-range interactions represent the most relevant functional category associated with the observed gene downregulation in MUT NSCs (Figure 5D). mRNAs encoding important transcription factors, and signal transduction molecules, are significantly reduced in MUT cells; among these factors, SOCS3 is able to rescue long-term self-renewal in MUT NSC, when overexpressed in these cells.

SOX2 Enrichment in Pol II-Mediated Long-Range Interactions between Promoters and Epigenetic Enhancers in NSC

A relevant role for SOX2 in long-range interactions can be hypothesized on the basis of the following observations: distal interaction anchors are highly enriched in epigenetic enhancer marks (Figure 2) and are highly represented in enhancers active in transgenic zebrafish (Figure 4) and in forebrain VISTA enhancers (Visel et al., 2009) (see Figures 3, 4, and S5). In addition, SOX2-bound sites are highly enriched in regions marked by epigenetic enhancer signatures (Figure 2B); in particular,

(D) Coassociation scores (histograms, -$\log_{10} p$) calculated for genes (TPM >5) showing either reduced gene expression in MUT NSCs (red), or no relevant change (green), and the indicated categories of interactions detected in wTR1, wTR2, and wTR3 (see STAR Methods). DOWN_MUT, genes showing statistically significant expression reduction; DOWN_MUT NO_SIGN, genes showing moderate expression reduction; OTHERS, all other genes. The types of interactions are shown on the right; data for "SOX2 promoter target" are not significant.
See also Tables S5 and S8.

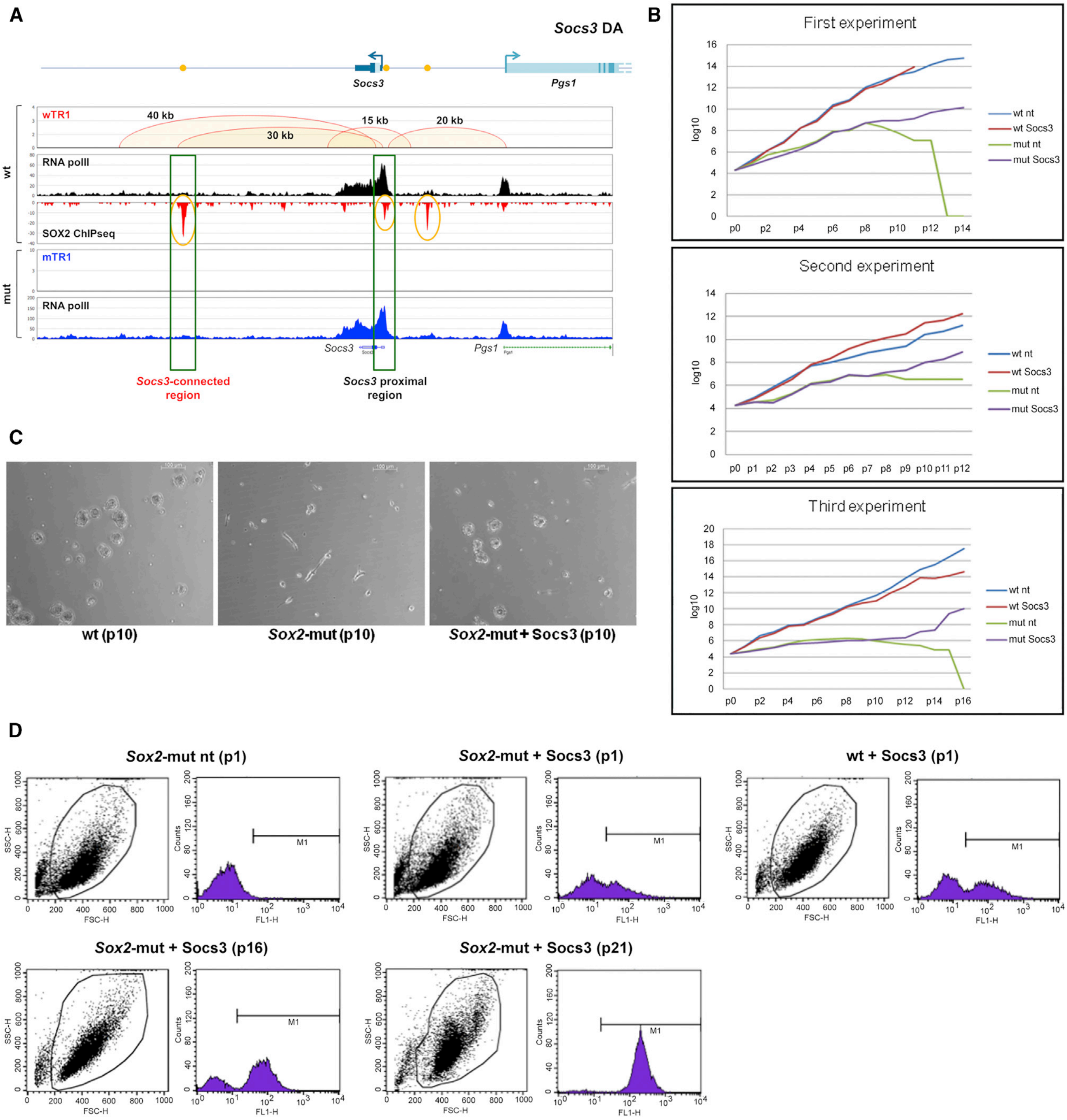

Figure 6. SOCS3 Re-expression in MUT NSCs Prevents NSC Exhaustion and Restores Self-Renewal

(A) Top: *Socs3* gene. ChIA-PET interactions, SOX2 peaks, and ChIA-PET reads in WT NSCs. Bottom: loss of interactions in *Sox2*-MUT NSCs.

(B) Growth curves of MUT NSCs, not transduced (MUT) or transduced (MUT *Socs3*) with a *Socs3*-GFP-expressing lentivirus, and of WT controls (WT or WT *Socs3*).

(C) Images (phase-contrast) of MUT or *Socs3*-transduced MUT NSCs 3 days after passage 12; neurospheres develop only from *Socs3*-transduced NSCs. For comparison, WT NSCs.

(D) FACS analysis (GFP) of MUT, WT, and MUT *Socs3* cells at the indicated passage number. With passaging, the fraction of GFP$^+$ NSCs progressively increases in MUT *Socs3* NSCs, eventually reaching 100%.

SOX2-bound epigenetically defined enhancer regions are much more represented than non-SOX2-bound regions in anchors (Figure 2E). Finally, upon ablation of *Sox2*, there is a reduction in interactions frequency, which is detected by ChIA-PET (Figures 1, 3, S3, and S5; Table 1) at large numbers of loci. It is thus possible to hypothesize that SOX2, perhaps in complex

with additional factors, may contribute to generate, or maintain, the network of interactions characteristic of NSCs.

A subset of P-P and P-E interactions is decreased in frequency in MUT cells. This might result from the loss of SOX2 from the interacting anchors (an appealing hypothesis) and the ensuing global chromatin conformation changes; an additional contribution to interaction loss might be represented by the transcriptional deregulation of many SOX2-controlled transcription factors (Tables S4 and S5) potentially contributing to interactions. Further, SOX2 interacts with several TFs, as well as with proteins involved in determining chromatin structure, such as NurD complex, SWI/SNF, CHD7, and SMRT/NCOR (Engelen et al., 2011), which are thus possible candidates for mediating such interactions.

Decreased Gene Expression in *Sox2* MUT Cells Is Significantly Correlated with Genes Whose Promoter Is Connected with an Enhancer Bound by SOX2 in WT Cells

The decreased transcription of ~1,000 genes (Tables S4 and S5) in *Sox2* MUT cells could in principle be ascribed to either loss of an effect of SOX2 on the gene promoter, or to loss of an effect on a connected enhancer. We demonstrate a predominant role of SOX2-bound enhancers versus non-SOX2-bound enhancers on the regulation of their connected genes (Figure 5D); in contrast, SOX2 binding to promoters is much less functionally relevant. Some interacting promoters may influence each other's activities (Li et al., 2012); our data, while not ruling out this model, suggest that, overall, the numerous P-P interactions detected in NSCs play a comparatively minor role relative to P-E interactions in SOX2-dependent regulation (Figures 5B and 5D). This is consistent with our observation (Zhang et al., 2013) that P-E interactions are more cell-type specific than P-P interactions, and is in agreement with the known cell-type specificity of SOX2 functions in neural cells. The predominant transcriptional effect of SOX2 at distal enhancer regions might be related either to an activating effect of SOX2 onto the connected promoter or to a stabilizing effect of SOX2 onto the interaction itself (as suggested by the decreased frequency of the interaction upon *Sox2* ablation), or both.

The role of interactions in controlling gene activity has been addressed by the knockout of genes encoding CTCF or Cohesin components in regulating chromatin interactions in cell lines. Only moderate transcriptional deregulation was observed in connection with widespread and deep changes in long-range interactions (Merkenschlager and Odom, 2013; Rao et al., 2017; Schwarzer et al., 2017); these proteins, however, are thought to act primarily as architectural proteins (Phillips-Cremins et al., 2013), in contrast to the well-established role of SOX2 as a transcription factor. During completion of this manuscript, the transcription factor YY1 was identified as a mediator of promoter-enhancer interactions in embryonic stem cells (Weintraub et al., 2017). YY1, contrary to SOX2, is ubiquitously expressed and acquires cell-type specificity of binding to DNA thanks to RNA and other undefined factors (Weintraub et al., 2017). Intriguingly, in neural progenitor cells (NPCs), YY1 is bound to a large subset of NPC-specific promoter-enhancer interactions (Beagan et al., 2017). It will be interesting to ask whether SOX2 and YY1 may functionally interact at this level.

Sox2 Loss Does Not Substantially Alter Epigenetic Enhancer Marks

While SOX2 is bound to a very large proportion of epigenetic enhancers in NSC and plays an important role in the regulation of a subset of genes, epigenetic marks on enhancers are not lost in *Sox2* MUT NSCs (Figure S4). This might be explained by the fact that no gene is completely silenced in the absence of SOX2, and interactions might be decreased, but not completely lost. Additionally, *Sox2* was ablated at a stage (E11.5) when specific EM might already have been established within NSCs; SOX2 might be initially important in determining the transition from an ectodermic cell to a NSC, and thus the establishment of proper chromatin EM, but might not be required afterward for their maintenance. These observations dissociate the presence of an epigenetic EM from the actual presence of a critical transcription factor on interacting promoter-enhancer complexes.

SOX2 Loss Affects the Activity of Key Genes Relevant to Cell Proliferation Control

Sox2 is expressed in NSCs throughout life (Zappone et al., 2000) and is essential for NSC maintenance in culture and *in vivo* in the hippocampus (Favaro et al., 2009). It is unknown which genes downstream to *Sox2* mediate its function in long-term NSC maintenance. *Socs3*, a highly connected gene (Figures 1 and 6) is strongly downregulated in *Sox2* MUT NSCs. *Socs3* transduction of a proportion of Sox2 MUT cells leads to a slow but progressive increase of the growth rate of the culture, with eventual recovery of a population of actively growing cells, providing evidence for a crucial role of *Socs3* in *Sox2*-dependent NSC maintenance (Figure 6). Interestingly, several additional genes (*c-fos*, *Jun*, *JunB*, *Btg2*, *Egr1*, and *Egr2*), encoding well-known regulators of cell proliferation, are expressed at high levels in WT NSCs and are substantially downregulated in *Sox2* MUT cells (Tables S4 and S5). These genes show multiple promoter-enhancer interactions in WT NSCs (not shown and Table S5) and might be part of a network of interacting genes required, together with *Socs3*, for optimal *Sox2*-dependent maintenance of NSCs. Hippocampal defects observed in *Sox2* MUT mice have been related to defects in NSCs (Favaro et al., 2009). The discovery of mediators of *Sox2* function in NSCs may be relevant to the understanding of *in vivo* defects.

Many Mouse Homologs of Genes Affected in Neurodevelopmental Brain Diseases Are Involved in Long-Range Interactions with Distant Regions Carrying Enhancer Marks

Thousands of polymorphisms in non-coding elements in man may be linked to brain disease or neurodevelopmental disorders (Nord et al., 2015). In NSCs, between ca. 1,750 and 3,500 expressed genes (Figure 5B, wTR1, wTR2, and wTR3) present long-range promoter-enhancer connections; the comparison of the regulatory elements that we identified in mouse with conserved orthologous sequences in man may allow identification of genes regulated by such enhancers, which might be dysfunctional in individuals carrying mutations at these elements.

Interestingly, the mouse homologs of several genes known to be involved in human neural disease show SOX2-bound

interactions (Figures 3B–3D and S5). A SOX2-bound neural enhancer within the *Akt3* gene is connected to the *Zbtb18* (*ZFP238* in man) TF gene (Figure 3B), whose mutation causes microcephaly in man and mouse (de Munnik et al., 2014); in man, deletions including *AKT3*, or translocations separating *AKT3* from *ZFP238* (Boland et al., 2007) (Figure 3B) also cause microcephaly. Both *Gpr56*, a gene whose promoter is bound by SOX2, and *Arid1a* (Figure 3) are connected to distant SOX2-bound epigenetic enhancers; mutation of the *GPR56* promoter causes structural neocortical abnormalities and *ARID1A* mutation is responsible for intellectual disability (Bae et al., 2014; Lee and Young, 2013, and references therein). The mutation of *SOX3* (see its enhancer in Figure 4) causes mental retardation and hypothalamic-pituitary defects (Laumonnier et al., 2002). Table S6 (see also Figure S5) lists additional genes involved in long-range interactions (many of which are SOX2-bound), whose human homologs are affected in neurodevelopmental disorders. In particular, Table S6 includes a large proportion of genes mutated in primary recessive microcephaly, severe intellectual disability, and eye disease. Significantly, the pathology of *SOX2* mutant patients includes brain (mainly hippocampal) abnormalities, some degree of mental retardation, and eye defects (Ragge et al., 2005; Sisodiya et al., 2006); microcephaly and some of the pathology observed in humans are also prominent in mouse Sox2 mutants (Favaro et al., 2009; Ferri et al., 2004, 2013). Our data suggest that some of the genes showing connections in Table S5 might play a role in human and mouse *Sox2*-dependent pathology; additionally, it might be interesting to search for mutations of the connected enhancers in human diseases such as intellectual disability and microcephaly.

STAR★METHODS

Detailed methods are provided in the online version of this paper and include the following:

- KEY RESOURCES TABLE
- CONTACT FOR REAGENTS AND RESOURCE SHARING
- EXPERIMENTAL MODEL AND SUBJECT DETAIL
  - Animals
  - Cell lines, primary cultures, microbe strains
- METHOD DETAILS
  - Cultures of neural stem/progenitor cells from wild-type and Sox2-deleted mouse P0 forebrain
  - ChIA-PET experiments
  - ChIA-PET data analyses
- CHIP-SEQ ANALYSES
  - SOX2 ChIPseq
  - H3K27Ac and H3K4me1 ChIPseq
  - Analysis of histone modifications colocalization by ChromHMM
  - Enrichment of SOX2-bound sites within H3K27Ac-enriched regions
  - Zebrafish transgenesis
  - RNA-Seq analysis
  - Co-association scores
  - Socs3 transduction in NSC
- DATA AND SOFTWARE AVAILABILITY
  - Accession numbers

SUPPLEMENTAL INFORMATION

Supplemental Information can be found with this article online at https://doi.org/10.1016/j.stem.2019.02.004.

ACKNOWLEDGMENTS

We thank Daofeng Li and Ting Wang (Washington University, http://wang.wustl.edu/) for helping us host and visualize our genomic data on the WashU browser. We thank Dr. Tae-Kyung Kim for the *c-fos* enhancers. Research reported in this publication was supported by Telethon, AIRC, Regione Lombardia ASTIL, Fondazione Cariplo, ERANET ImprovVision (NEURON8-Full-815-091 to S.K.N.), MINECO (BFU2013-43213-P and BFU2016-75412-R with FEDER support), CIBERER, ISCIII, ERANET/PCIN-2015-176-C02-01, an Institutional Grant from Fundación Ramon Areces and Banco Santander (to P.B.), the 4DN (U54 DK107967), ENCODE Consortia (UM1 HG009409 to C.-L.W.), NCI (P30CA034196 to C.-L.W. and C.Y.N.), the Francis Crick Institute (which receives its core funding from Cancer Research UK; FC0010089), the UK Medical Research Council (FC0010089), the Wellcome Trust (FC0010089 to F.G. and B.M.), and the UK Biotechnology and Biological Sciences Research Council (BB/K005316/1 to F.G.). J.B. was supported by short-term fellowships from EMBO and Fondazione Cariplo for two stays in Madrid.

AUTHOR CONTRIBUTIONS

C.-L.W. and S.K.N. designed the study. S.K.N., C-L.W., G.P., S.O., F.G., M.C., and P.B. discussed the results and wrote the manuscript with input from all other authors. R.F. and M.P. prepared NSCs for all chromatin studies. Y.Z. and H.T. performed and analyzed the TR2 and TR3 ChIA-PET data. C.-H.W. analyzed TR1 ChIA-PET data. C.Y.N. performed RNA-seq. B.M. and M.V. did Sox2 and histone marks ChIP-seq. P.R. and J.M. did initial RNA-seq work. G.P. and F.Z. planned and performed bioinformatics and biostatistics analyses. R.F., C.B., J.B., and S.M. made DNA constructs and transfections. J.B., M.J.C., and N.T. performed zebrafish studies under the guidance of P.B. M.P. did the MUT NSC rescue experiments.

DECLARATION OF INTERESTS

The authors declare no competing interests.

Received: January 30, 2018
Revised: July 16, 2018
Accepted: February 6, 2019
Published: March 7, 2019

REFERENCES

Avilion, A.A., Nicolis, S.K., Pevny, L.H., Perez, L., Vivian, N., and Lovell-Badge, R. (2003). Multipotent cell lineages in early mouse development depend on SOX2 function. Genes Dev. *17*, 126–140.

Bae, B.I., Tietjen, I., Atabay, K.D., Evrony, G.D., Johnson, M.B., Asare, E., Wang, P.P., Murayama, A.Y., Im, K., Lisgo, S.N., et al. (2014). Evolutionarily dynamic alternative splicing of GPR56 regulates regional cerebral cortical patterning. Science *343*, 764–768.

Beagan, J.A., Duong, M.T., Titus, K.R., Zhou, L., Cao, Z., Ma, J., Lachanski, C.V., Gillis, D.R., and Phillips-Cremins, J.E. (2017). YY1 and CTCF orchestrate a 3D chromatin looping switch during early neural lineage commitment. Genome Res. *27*, 1139–1152.

Bessa, J., Tena, J.J., de la Calle-Mustienes, E., Fernández-Miñán, A., Naranjo, S., Fernández, A., Montoliu, L., Akalin, A., Lenhard, B., Casares, F., and Gómez-Skarmeta, J.L. (2009). Zebrafish enhancer detection (ZED) vector: a new tool to facilitate transgenesis and the functional analysis of cis-regulatory regions in zebrafish. Dev. Dyn. *238*, 2409–2417.

Boland, E., Clayton-Smith, J., Woo, V.G., McKee, S., Manson, F.D., Medne, L., Zackai, E., Swanson, E.A., Fitzpatrick, D., Millen, K.J., et al. (2007). Mapping of deletion and translocation breakpoints in 1q44 implicates the serine/threonine kinase AKT3 in postnatal microcephaly and agenesis of the corpus callosum. Am. J. Hum. Genet. *81*, 292–303.

Cantone, I., and Fisher, A.G. (2011). Unraveling epigenetic landscapes: the enigma of enhancers. Cell Stem Cell *8*, 128–129.

Cao, F., Hata, R., Zhu, P., Ma, Y.J., Tanaka, J., Hanakawa, Y., Hashimoto, K., Niinobe, M., Yoshikawa, K., and Sakanaka, M. (2006). Overexpression of SOCS3 inhibits astrogliogenesis and promotes maintenance of neural stem cells. J. Neurochem. *98*, 459–470.

Creyghton, M.P., Cheng, A.W., Welstead, G.G., Kooistra, T., Carey, B.W., Steine, E.J., Hanna, J., Lodato, M.A., Frampton, G.M., Sharp, P.A., et al. (2010). Histone H3K27ac separates active from poised enhancers and predicts developmental state. Proc. Natl. Acad. Sci. USA *107*, 21931–21936.

de Laat, W., and Duboule, D. (2013). Topology of mammalian developmental enhancers and their regulatory landscapes. Nature *502*, 499–506.

de Munnik, S.A., García-Miñaúr, S., Hoischen, A., van Bon, B.W., Boycott, K.M., Schoots, J., Hoefsloot, L.H., Knoers, N.V., Bongers, E.M., and Brunner, H.G. (2014). A de novo non-sense mutation in ZBTB18 in a patient with features of the 1q43q44 microdeletion syndrome. Eur. J. Hum. Genet. *22*, 844–846.

Engelen, E., Akinci, U., Bryne, J.C., Hou, J., Gontan, C., Moen, M., Szumska, D., Kockx, C., van Ijcken, W., Dekkers, D.H., et al. (2011). Sox2 cooperates with Chd7 to regulate genes that are mutated in human syndromes. Nat. Genet. *43*, 607–611.

Ernst, J., and Kellis, M. (2012). ChromHMM: automating chromatin-state discovery and characterization. Nat. Methods *9*, 215–216.

Esteve, P., Lopez-Rios, J., and Bovolenta, P. (2004). SFRP1 is required for the proper establishment of the eye field in the medaka fish. Mech. Dev. *121*, 687–701.

Favaro, R., Valotta, M., Ferri, A.L., Latorre, E., Mariani, J., Giachino, C., Lancini, C., Tosetti, V., Ottolenghi, S., Taylor, V., and Nicolis, S.K. (2009). Hippocampal development and neural stem cell maintenance require Sox2-dependent regulation of Shh. Nat. Neurosci. *12*, 1248–1256.

Ferri, A.L., Cavallaro, M., Braida, D., Di Cristofano, A., Canta, A., Vezzani, A., Ottolenghi, S., Pandolfi, P.P., Sala, M., DeBiasi, S., et al. (2004). Sox2 deficiency causes neurodegeneration and impaired neurogenesis in the adult mouse brain. Development *131*, 3805–3819.

Ferri, A., Favaro, R., Beccari, L., Bertolini, J., Mercurio, S., Nieto-Lopez, F., Verzeroli, C., La Regina, F., De Pietri Tonelli, D., Ottolenghi, S., et al. (2013). Sox2 is required for embryonic development of the ventral telencephalon through the activation of the ventral determinants Nkx2.1 and Shh. Development *140*, 1250–1261.

Fishman, M.C., Stainier, D.Y., Breitbart, R.E., and Westerfield, M. (1997). Zebrafish: genetic and embryological methods in a transparent vertebrate embryo. Methods Cell Biol. *52*, 67–82.

Francipane, M.G., Eterno, V., Spina, V., Bini, M., Scerrino, G., Buscemi, G., Gulotta, G., Todaro, M., Dieli, F., De Maria, R., and Stassi, G. (2009). Suppressor of cytokine signaling 3 sensitizes anaplastic thyroid cancer to standard chemotherapy. Cancer Res. *69*, 6141–6148.

Gonçalves, J.T., Schafer, S.T., and Gage, F.H. (2016). Adult Neurogenesis in the Hippocampus: From Stem Cells to Behavior. Cell *167*, 897–914.

Gorkin, D.U., Leung, D., and Ren, B. (2014). The 3D genome in transcriptional regulation and pluripotency. Cell Stem Cell *14*, 762–775.

Hagey, D.W., Zaouter, C., Combeau, G., Lendahl, M.A., Andersson, O., Huss, M., and Muhr, J. (2016). Distinct transcription factor complexes act on a permissive chromatin landscape to establish regionalized gene expression in CNS stem cells. Genome Res. *26*, 908–917.

Langmead, B. (2010). Aligning short sequencing reads with Bowtie. Curr. Protoc. Bioinformatics *Chapter 11*. Unit 11.17.

Laumonnier, F., Ronce, N., Hamel, B.C., Thomas, P., Lespinasse, J., Raynaud, M., Paringaux, C., Van Bokhoven, H., Kalscheuer, V., Fryns, J.P., et al. (2002). Transcription factor SOX3 is involved in X-linked mental retardation with growth hormone deficiency. Am. J. Hum. Genet. *71*, 1450–1455.

Lee, T.I., and Young, R.A. (2013). Transcriptional regulation and its misregulation in disease. Cell *152*, 1237–1251.

Li, B., and Dewey, C.N. (2011). RSEM: accurate transcript quantification from RNA-Seq data with or without a reference genome. BMC Bioinformatics *12*, 323.

Li, H., and Durbin, R. (2009). Fast and accurate short read alignment with Burrows-Wheeler transform. Bioinformatics *25*, 1754–1760.

Li, G., Fullwood, M.J., Xu, H., Mulawadi, F.H., Velkov, S., Vega, V., Ariyaratne, P.N., Mohamed, Y.B., Ooi, H.S., Tennakoon, C., et al. (2010). ChIA-PET tool for comprehensive chromatin interaction analysis with paired-end tag sequencing. Genome Biol. *11*, R22.

Li, G., Ruan, X., Auerbach, R.K., Sandhu, K.S., Zheng, M., Wang, P., Poh, H.M., Goh, Y., Lim, J., Zhang, J., et al. (2012). Extensive promoter-centered chromatin interactions provide a topological basis for transcription regulation. Cell *148*, 84–98.

Lodato, M.A., Ng, C.W., Wamstad, J.A., Cheng, A.W., Thai, K.K., Fraenkel, E., Jaenisch, R., and Boyer, L.A. (2013). SOX2 co-occupies distal enhancer elements with distinct POU factors in ESCs and NPCs to specify cell state. PLoS Genet. *9*, e1003288.

Mateo, J.L., van den Berg, D.L., Haeussler, M., Drechsel, D., Gaber, Z.B., Castro, D.S., Robson, P., Lu, Q.R., Crawford, G.E., Flicek, P., et al. (2015). Characterization of the neural stem cell gene regulatory network identifies OLIG2 as a multifunctional regulator of self-renewal. Genome Res. *25*, 41–56.

Merkenschlager, M., and Odom, D.T. (2013). CTCF and cohesin: linking gene regulatory elements with their targets. Cell *152*, 1285–1297.

Mumbach, M.R., Rubin, A.J., Flynn, R.A., Dai, C., Khavari, P.A., Greenleaf, W.J., and Chang, H.Y. (2016). HiChIP: efficient and sensitive analysis of protein-directed genome architecture. Nat. Methods *13*, 919–922.

Nord, A.S., Pattabiraman, K., Visel, A., and Rubenstein, J.L.R. (2015). Genomic perspectives of transcriptional regulation in forebrain development. Neuron *85*, 27–47.

Okuda, Y., Ogura, E., Kondoh, H., and Kamachi, Y. (2010). B1 SOX coordinate cell specification with patterning and morphogenesis in the early zebrafish embryo. PLoS Genet. *6*, e1000936.

Paulsen, J., Rodland, E.A., Holden, L., Holden, M., and Hovig, E. (2014).). A statistical model of ChIA-PET data for accurate detection of chromatin 3D interactions. Nucleic Acids Res. *42*, e143.

Phillips-Cremins, J.E., Sauria, M.E., Sanyal, A., Gerasimova, T.I., Lajoie, B.R., Bell, J.S., Ong, C.T., Hookway, T.A., Guo, C., Sun, Y., et al. (2013). Architectural protein subclasses shape 3D organization of genomes during lineage commitment. Cell *153*, 1281–1295.

Ragge, N.K., Lorenz, B., Schneider, A., Bushby, K., de Sanctis, L., de Sanctis, U., Salt, A., Collin, J.R., Vivian, A.J., Free, S.L., et al. (2005). SOX2 anophthalmia syndrome. Am. J. Med. Genet. A. *135*, 1–7, discussion 8.

Rao, S.S.P., Huang, S.C., Glenn St Hilaire, B., Engreitz, J.M., Perez, E.M., Kieffer-Kwon, K.R., Sanborn, A.L., Johnstone, S.E., Bascom, G.D., Bochkov, I.D., et al (2017). Cohesin Loss Eliminates All Loop Domains. Cell *171*, 305–320.

Rivera, C.M., and Ren, B. (2013). Mapping human epigenomes. Cell *155*, 39–55.

Sanyal, A., Lajoie, B.R., Jain, G., and Dekker, J. (2012). The long-range interaction landscape of gene promoters. Nature *489*, 109–113.

Schwarzer, W., Abdennur, N., Goloborodko, A., Pekowska, A., Fudenberg, G., Loe-Mie, Y., Fonseca, N.A., Huber, W., H Haering, C., Mirny, L., and Spitz, F. (2017). Two independent modes of chromatin organization revealed by cohesin removal. Nature *551*, 51–56.

Sisodiya, S.M., Ragge, N.K., Cavalleri, G.L., Hever, A., Lorenz, B., Schneider, A., Williamson, K.A., Stevens, J.M., Free, S.L., Thompson, P.J., et al. (2006). Role of SOX2 mutations in human hippocampal malformations and epilepsy. Epilepsia *47*, 534–542.

Takahashi, K., and Yamanaka, S. (2006). Induction of pluripotent stem cells from mouse embryonic and adult fibroblast cultures by defined factors. Cell *126*, 663–676.

Tarazona, S., Furió-Tarí, P., Turrà, D., Pietro, A.D., Nueda, M.J., Ferrer, A., and Conesa, A. (2015). Data quality aware analysis of differential expression in RNA-seq with NOISeq R/Bioc package. Nucleic Acids Res. *43*, e140.

Vermunt, M.W., Tan, S.C., Castelijns, B., Geeven, G., Reinink, P., de Bruijn, E., Kondova, I., Persengiev, S., Bontrop, R., Cuppen, E., et al.; Netherlands Brain Bank (2016). Epigenomic annotation of gene regulatory alterations during evolution of the primate brain. Nat. Neurosci. *19*, 494–503.

Visel, A., Blow, M.J., Li, Z., Zhang, T., Akiyama, J.A., Holt, A., Plajzer-Frick, I., Shoukry, M., Wright, C., Chen, F., et al. (2009). ChIP-seq accurately predicts tissue-specific activity of enhancers. Nature *457*, 854–858.

Weintraub, A.S., Li, C.H., Zamudio, A.V., Sigova, A.A., Hannett, N.M., Day, D.S., Abraham, B.J., Cohen, M.A., Nabet, B., Buckley, D.L., et al. (2017). YY1 Is a Structural Regulator of Enhancer-Promoter Loops. Cell *171*, 1573–1588.

Yang, T., Zhang, F., Yardımcı, G.G., Song, F., Hardison, R.C., Noble, W.S., Yue, F., and Li, Q. (2017). HiCRep: assessing the reproducibility of Hi-C data using a stratum-adjusted correlation coefficient. Genome Res. *27*, 1939–1949.

Zappone, M.V., Galli, R., Catena, R., Meani, N., De Biasi, S., Mattei, E., Tiveron, C., Vescovi, A.L., Lovell-Badge, R., Ottolenghi, S., and Nicolis, S.K. (2000). Sox2 regulatory sequences direct expression of a (beta)-geo transgene to telencephalic neural stem cells and precursors of the mouse embryo, revealing regionalization of gene expression in CNS stem cells. Development *127*, 2367–2382.

Zhang, Y., Liu, T., Meyer, C.A., Eeckhoute, J., Johnson, D.S., Bernstein, B.E., Nusbaum, C., Myers, R.M., Brown, M., Li, W., and Liu, X.S. (2008). Model-based analysis of ChIP-Seq (MACS). Genome Biol. *9*, R137.

Zhang, Y., Wong, C.H., Birnbaum, R.Y., Li, G., Favaro, R., Ngan, C.Y., Lim, J., Tai, E., Poh, H.M., Wong, E., et al. (2013). Chromatin connectivity maps reveal dynamic promoter-enhancer long-range associations. Nature *504*, 306–310.

Zhu, J., Adli, M., Zou, J.Y., Verstappen, G., Coyne, M., Zhang, X., Durham, T., Miri, M., Deshpande, V., De Jager, P.L., et al. (2013). Genome-wide chromatin state transitions associated with developmental and environmental cues. Cell *152*, 642–654.

STAR★METHODS

KEY RESOURCES TABLE

REAGENT or RESOURCE	SOURCE	IDENTIFIER
Antibodies		
Anti-RNAPII monoclonal antibody (TR1)	Covance	Cat# 8WG16; RRID: AB_10013665
Goat anti-SOX2 antibody	Santa Cruz	Cat# sc-17320; RRID: AB_2286684
Anti-H3K27ac antibody	Abcam	Cat# Ab4729; RRID: AB_2118291
Anti-H3K4me1 antibody	Abcam	Cat# Ab8895; RRID: AB_306847
Anti-RNAPII monoclonal antibody (TR2, TR3)	Biolegend	920102; RRID: AB_2565318
Bacterial and Virus Strains		
TWEEN lentiviral vector expressing CMV-SOCS3 and PGK-GFP	Francipane et al., 2009	N/A
Chemicals, Peptides, and Recombinant Proteins		
Protein G	Invitrogen	10004D
Illumina Nextera DNA sample prep kit	Illumina	FC-121-1030
Illumina Nextera indexes	Illumina	FC-121-1011
EGS	Thermo	21565
Triton X-100	Sigma-Aldrich	T8787-100ml
dATPI	NEB	N0440S
Klenow (3′-5′ EXO-)-1 000	NEB	M0212L
T4 DNA ligase	NEB	M0202L
AluI	NEB	R0137L
BSA	NEB	B9000S
10% SDS	Ambion	AM9822
Formaldehyde	Sigma	F8775
5 × ligation buffer	NEB	B6058S
Glycine	Sigma	G8898
Critical Commercial Assays		
TruSeq ChIP Sample Prep Kit	Illumina	Cat# IP-2020-1012 or 1024
pCR8/GW/TOPO® TA Cloning® Kit	Thermo Fisher	Cat# K250020
Clonase™-assisted, Gateway® technology	Invitrogen	Cat# 12535-019, 12535-027
Truseq Stranded mRNA Sample Preparation Kit	Illumina	Cat# RS-122-2101/2/3
Deposited Data		
Raw and analyzed data	This paper	GEO: GSE90561
Experimental Models: Cell Lines		
Ex-vivo cultured NSCs from P0 mouse forebrains	Favaro et al., 2009	N/A
Experimental Models: Organisms/Strains		
Mouse: Sox2fl/fl;nestin-Cre conditional Sox2 mutants	Favaro et al., 2009	EMMA ID (Sox2flox): EM:07995
Oligonucleotides		
PCR primers for anchor amplification are in Methods (Methods S1 primers for anchor amplification)	N/A	N/A
Sox2-specific morpholino	Okuda et al., 2010	N/A
Recombinant DNA		
Zebrafish Enhancer Detection vector	Bessa et al., 2009	N/A
Software and Algorithms		
Bowtie version 0.12.5 (for SOX2 ChIPseq)	Langmead, 2010	http://bowtie-bio.sourceforge.net
MACS version 2.0.9 (for SOX2 ChIPseq)	Zhang et al., 2008	N/A
Bowtie version 1.1.0 (for H3K27ac/H3K4me1 ChIPseq)	N/A	http://bowtie-bio.sourceforge.net
MACS2 (for H3K27ac/H3K4me1 ChIPseq)	N/A	N/A

(*Continued on next page*)

Continued		
REAGENT or RESOURCE	SOURCE	IDENTIFIER
RSEM software package version 1.17	Li and Dewey, 2011	N/A
Noiseq package	Tarazona et al., 2015	N/A
ChIA-PET Tools	https://github.com/cheehongsg/CPU	N/A
ChiaSigScaled	https://github.com/cheehongsg/ChiaSigScaled	N/A

CONTACT FOR REAGENTS AND RESOURCE SHARING

Further information and requests for resources and reagents should be directed to and will be fulfilled by the Lead Contact Silvia K. Nicolis (silvia.nicolis@unimib.it).

EXPERIMENTAL MODEL AND SUBJECT DETAIL

Animals

Sox2 conditional mutant mice

Mutant and wild-type mice were sacrificed at P0, to obtain forebrains for NSC cultures (sex was indifferent). *Sox2* deletion was obtained by breeding *Sox2*^{flox} mutant mice to *nestinCre* transgenic mice (as in Favaro et al., 2009). The experiments were approved by the Italian Ministery of Health as conforming to the relevant regulatory standards.

Zebrafish

AB and *tupl* wild-type zebrafish strains were maintained and bred according to standard procedures (Fishman et al, 1997). All experiments conform to the guidelines from the European Community Directive and the Spanish legislation for the experimental use of animals.

Cell lines, primary cultures, microbe strains

Primary ex-vivo neural stem/progenitor cell cultures

P0 brain-derived NSC cultures were obtained from dissected telencephalon of wild-type and *Sox2*-deleted mice, and grown, as described in Favaro et al. (2009) and Zhang et al. (2013), see Method Details section below.

Microbe strains

Standard cloning procedures were carried out in *E. coli* TOP ten and DH5alpha.

Lentivirus packaging cell lines

Packaging of the *Socs3*-expressing lentivirus was performed in 293T cells.

METHOD DETAILS

Cultures of neural stem/progenitor cells from wild-type and Sox2-deleted mouse P0 forebrain

After forebrain dissection and cell dissociation, we plated cells in 25 mL flasks and cultured them to expand their number in complete medium (2% (vol/vol) B27 in DMEM F12 with Glutamax), supplemented with 10 ng/ml EGF, 10 ng/ml of basic fibroblast growth factor (FGF) with 0.2% (vol/vol) heparin. We cultured wild-type and mutant cells in parallel for two initial passages (until sphere formation was detected, 3-7 days, normally 4 days) (Favaro et al., 2009), followed by sphere dissociation and further expansion in the presence of EGF, but not bFGF, for 3-5 more passages (4 days), as described in Zhang et al. (2013), for optimal maintenance of mutant NSC (Favaro et al., 2009). Cell passaging involved 0.25% trypsin treatment for 5 min, followed by block with 1 mg/ml trypsin inhibitor (Sigma T6522) for 5 min; neurospheres were then mechanically dissociated by pipetting and cells were seeded at 80,000 per ml in T150 flasks.

After obtaining appropriate numbers of wild-type and mutant cells, we started collecting part of the neurospheres at each passage, continuing the culture of the remaining cells as long as the growth of mutant cells was comparable to that of wild-type cells. In order to proceed to ChIA-PET and ChIP-seq experiments, we mechanically dissociated and crosslinked the neurospheres as described below.

ChIA-PET experiments

ChIA-PET experiments for TR1were performed as described (Zhang et al., 2013), using pooled NSC from four wild-type and six mutant brains from littermates. Wild-type and mutant cultures were processed in parallel. wTR1 data are from Zhang et al. (2013); mTR1 data, present paper. Neurospheres were cross-linked by standard formaldehyde treatment and the pellets were then snap-frozen in nitrogen. The crosslinked cells were lysed to release the chromatin–DNA complexes followed by fragmentation to an average size of 300 base pairs (bp). The sonicated chromatin–DNA complexes were incubated with the Pol II monoclonal

antibody-coated magnetic Protein G beads (8WG16, Covance). To determine the ChIP quality, a small portion of ChIP DNA was eluted for quantitative PCR (qPCR) analysis. For ChIA-PET library construction, ChIP-enriched chromatin complexes were divided into two aliquots. To distinguish the intramolecular proximity ligation products from the chimeras resulting from non-specific inter-molecular ligations, two different barcoded biotinylated half-linkers (linker A and linker B) were ligated to the ends of polished bead-bound-DNA fragments and used to join the juxtaposed chromatin regions. The half-linker-ligated chromatin–DNA fragments were pooled for phosphorylation and proximity-based circularization. MmeI was subsequently used to release the paired-end tags (PETs). The full-length linkers AA/BB resulting from intra-molecular circularization were considered to be non-chimeric PETs. Conversely, the chimeric full linkers AB/BA resulting from intermolecular ligation were considered to be ligation noises. The biotin-labeled PET constructs were amplified and subjected to sequencing analysis.

For the *in situ* Pol II ChIA-PET 10 million formaldehyde crosslinked cells for each analysis (wTR2, wTR3 and mTR2, mTR3), (cells grown from individual brains, as above) were suspended with 100 μL 0.55% SDS and incubated at RT for 10 min, 62°C for 10 min and 37°C for 10 min to permeabilize nuclei, which was followed by addition of 25 μL 20% Triton X-100, incubation of 30 min at 37°C to quench SDS, followed by addition of 50 μL of blunt-end four-cutter AluI (cat# R0137L, NEB), 50 μL 10 × CutSmart buffer and 275 μL H2O and incubation at 37°C overnight for *in situ* digestion. After pelleting and washing once with 1 mL 1 × CutSmart buffer, the nuclei were suspended in 500 μL A tailing solution composed of 50 μL 10 × CutSmart buffer, 10 μL BSA (cat#B9000S, NEB), 10 μL 10 mM dATP (cat#N0440S, NEB), 10 μL Klenow (3′-5′ exo-) (cat#M0202L, NEB), and 420 μL H2O and incubated at RT for 1 hr. The *in situ* proximity ligation with biotinylated bridge linker was performed by adding 200 μL 5 × ligation buffer (cat#B6058S, NEB), 6 μL Bridge linker (200 ng/ul), 10 μL T4 DNA ligase (cat#M0202L, NEB) and 284 μL H2O and incubating at 16°C overnight. The nuclei with *in situ* proximity ligation were then subjected to sonication and chromatin immunoprecipitation with anti-Pol II antibody (8WG16, cat# 920102, Biolegend, San Diego, CA), Tn5 tagmentation, and biotin selection; PETs were amplified by PCR and sequenced.

ChIA-PET data analyses

The TR1 sequence data generated from the original ChIA-PET protocol were analyzed using ChIA-PET tool (Li et al., 2010). In brief, non-redundant PET sequence reads were first analyzed for linker barcode composition and non-chimeric PETs were used for further analysis. Next, the linker sequences were trimmed, and the PET sequences were mapped to the mouse reference genome (mm9) with 1 mismatch allowed. The PETs with genomic locations from both head and tail tags within 2 bp were merged to further filter the redundancy arising from clonal PCR amplification. Based on the mapping coordinates, the specific-ligation PETs were used for further classification as inter-chromosomal, intra-chromosomal and self-ligation PETs. Inter-chromosomal PETs were defined as both the head and tail of the PETs uniquely mapped onto different chromosomes. To define highly reliable interaction clusters, we adopted the false discovery rate (FDR) of the hyper-geometric model. Such model takes into consideration the tag counts from both anchor regions and the sequencing depth to determine reliable, i.e., significant, interactions. A FDR cutoff 0.05 was used. Finally, we performed a random shuffling simulation to evaluate the correlation between noise level and PET cluster counts. The simulation broke down the pairing relationship of different PET clusters and the tags were randomly paired to generate simulated PETs. We further compared the interaction numbers between simulations versus our experimental data and determined the noise level (number of simulated clusters / number of real clusters) for the PET-2+ (PET cluster with 2 counts and above) to be 1.2%. Therefore, we chose the PET cluster > = 2 to keep the noise level low and singletons (PET = 1) were considered as noise. For TR2 and TR3 generated by the *in situ* ChIA-PET protocol, ChIA-PET data were processed with ChIA-PET Utilities, a scalable re-implementation of ChIA-PET tool. Briefly, sequencing adaptors incorporated during the tagmentation reaction in the library construction process were removed from the paired reads. Tags identified (> = 18bp) were mapped to mouse genome (mm9) using BWA alignment (Li and Durbin, 2009) and memarXiv:1303.3997 [q-bio.GN], https://arxiv.org/abs/1303.3997 according to their tag length. The duplicated pair-end tags arising from clonal PCR amplification were filtered and the uniquely mapped, non-redundant PETs were classified as inter-chromosomal (L tags and R tags mapped onto different chromosomes), intra-chromosomal (L tags and R tags mapped onto the same chromosome with genomic distance > 8Kb) and self-ligation PETs (L tags and R tags mapped onto the genome ≤ 8Kb). Multiple intra-chromosomal PETs whose respective ends were found within 1 Kb were then clustered as iPET-2, 3 … We further performed statistical assessment of the PET clusters interaction significance using ChiaSigScaled, a scalable re-implementation of ChiaSig (Paulsen et al., 2014). Interaction clusters with member size 3 and above (iPET 3+) and FDR < 0.05 were classified as significant interactions (Table 1, line 7). Among these, the interactions with Pol II binding at both anchors were further defined as Pol II-mediated interactions (Table 1, line 8). Next, the interactions were classified based on their anchors overlapped with gene models. Each anchor was annotated with the gene that overlapped at 1bp overlap. To classify each anchor, priority was given to promoter (P) region (defined as ± 2.5kb of TSS) followed by gene region (G). Anchors that do not overlap with any gene or promoter region were classified as intergenic (I). The interaction classification is just the combination of its anchors classification. To determine the reproducibility between TR2 and TR3, we adopted the method used in the Hi-C data (Yang et al., 2017) to determine the SCC, Stratum-adjusted Correlation Coefficient. To compute the SCC, ChIA-PET loops for each library were aggregated into 10-kb bin matrix. SCC score is computed with HiCrep method (hicrep library in R).

For each chromosome, the smoothing parameter was used as recommended (h = 3), with maximum distance 1 Mb. Because the methods used to fragment chromatins and generate tags for sequencing were different in TR1, TR1 was not included in the SCC analysis. As TR1 used sonication shearing and MmeI digestion while TR2 and TR3 used AluI digestion and Tn5 transposon-based tagmentation, the exact anchor locations defined by these two methods cannot be directly compared.

ChIP-seq ANALYSES

SOX2 ChIPseq

NSC from 6 wild-type forebrains, at P0 (Favaro et al., 2009), were independently grown, and analyzed by ChIPseq in duplicate. Individual cultures were pooled together, at a stage when neurospheres were still relatively small, and then divided into two aliquots: neurospheres were directly fixed for the first ChIP (''spheres'' ChIPseq), whereas single cells (resulting from dissociation of the same neurospheres) were used for the second ChIP (''singles'' ChIPseq) (see GEO).

Cells were fixed sequentially with di(N-succimidyl) glutarate and 1% formaldehyde in phosphate-buffered saline and then lysed, sonicated and immunoprecipitated as described previously (Mateo et al., 2015 and references therein). SOX2 was immunoprecipitated with 3mg of goat anti-SOX2 (Santa Cruz sc-17320).

DNA libraries were prepared from 10ng of immunoprecipitated DNA and 10ng of input DNA control, according to the standard Illumina ChIP-seq protocol. Libraries were sequenced with the Genome Analyzer IIx (Illumina). The raw reads were mapped to the mouse genome (mm9 including random chromosomes) with Bowtie (Langmead, 2010) version 0.12.5. We used MACS (Zhang et al., 2008) version 2.0.9 to define SOX2-bound regions (peaks). As this tool is very sensitive to the unbalanced number of reads in the real and the input set, we decided to reduce the larger input dataset to match the number of mapped reads in the smaller IP dataset by randomly downsampling reads, as described previously (Mateo et al., 2015).

By using default significance thresholds, this resulted in 18,359 SOX2-bound peak regions for the first (''spheres'') ChIPseq. Since fixation of whole neurospheres might not, in theory, be equally efficient for internal relative to external cells, we also performed another ChIP experiment (''singles'' ChIPseq). Read mapping and peak calling were performed with the same parameters used in the first experiment, producing 43,070 bound regions at the same significance thresholds. Of the first dataset (''spheres'' ChIPseq), the vast majority (15,985 peaks, 87%) were contained in the latter (''singles'' ChIPseq) peak list. Since also the 13% non-overlapping peaks for the first experiment showed enrichment in the corresponding loci in the second ChIPseq, even if below the ''peak detection threshold,'' we kept for all the subsequent analyses presented in the paper all the peaks returned by the first experiment (''spheres'').

The difference between ''spheres'' and ''singles'' ChIPseq appears to be due mainly to the presence in ''singles'' of numerous additional small peaks that have no (significant) corresponding peaks in the ''spheres'' sample, and may represent more marginal binding sites. Indeed, comparison of profiles between ''spheres'' and ''singles'' showed little differences between them, indicating that the main binding sites are very similar (data not shown).

Finally, over 50% of the ca. 18400 peaks observed in our forebrain NSC ''spheres'' are found also within the ca. 24800 peaks detected in a ChIPseq analysis of the NS-5 cell line (Mateo et al., 2015), an ES-derived NSC line that shows a general (nonforebrain-specific) neural phenotype. For these reasons, we used for subsequent analyses the data from ''spheres.''

H3K27Ac and H3K4me1 ChIPseq

NSC were derived from six forebrains of wild-type and 6 forebrains of *Sox2*-deleted mice, at P0 (Favaro et al., 2009). NSC were initially cultured individually, then pooled according to wild-type or mutant genotype to generate two independent pools. Each independent pool was divided in two parts, and used for ChIP-sequencing on H3K27ac or H3K4me1, as described previously (Vermunt et al., 2016). Neurospheres were dissociated and 4 million single cells were crosslinked with 1% formaldehyde for 10 minutes at room temperature. Reaction was quenched with 0.125 M Glycine, cells were washed with cold PBS and lysed according to Vermunt et al. (2016). Nuclei pellets were resuspended in 160 μL sonication buffer and divided over two microtubes for shearing in the Covaris S series with the following settings for 12 cycles of 60 s: intensity 3, duty cycle 20%, 200 cycles/bursts. Chromatin immunoprecipitation steps after sonication were performed as described previously (Vermunt et al., 2016) using 50 μL Dynal protein G beads that were preincubated with 5 μg Ab4729 (Abcam) for H3K27ac or 5 μg Ab8894 (Abcam) for H3K4me1. Whole cell extract of 4 million cells was split onto both antibodies, resulting in the use of 2 million cells per ChIP. Libraries were made using the Illumina Truseq DNA library protocol and sequencing was done at the MIT BioMicro Center (https://openwetware.org/wiki/BioMicroCenter). Obtained sequences were aligned onto the mm9 mouse genome assembly using Bowtie 1.1.0 (http://bowtie-bio.sourceforge.net) excluding reads that had more than 1 mismatch or that could map to multiple genomic locations. MACS2 was used for peak calling (p value threshold = 10^{-5}, extsize = 400, local lambda = 100,000) and narrowpeaks were extended to a minimum of 2000 basepairs (bps) to match peak resolution. Overlapping enriched regions were merged and were considered promoters when located within 1000 bps from annotated mm9 transcriptional start sites (TSSs) and considered putative distal enhancers when located more than 1000 bps away from TSSs.

Analysis of histone modifications colocalization by ChromHMM

Co-localization of histone modifications was performed with ChromHMM version 1.4. (Ernst and Kellis, 2012). Briefly, the software partitions the genome into non overlapping segments of 200 bps. Then, given a set of histone modification ChIP-Seq experiments, associates to each segment each of the histone modifications if the number of reads mapping in the segment can be considered to be enriched according to a random background Poisson distribution. Then, given a number of states as input, it evaluates the co-occurrence of histone modifications in the genome segments, building a model in which each of the states is characterized by a given combination of modifications.

The program was run setting a different number of states, and by processing either wild-type (WT) samples alone and mutant (MUT) samples alone, and on both WT and MUT samples combined. In every setting, the model recovered consistently four main

states, corresponding to the joint presence of H3K27ac and H3K4me1, either modification alone, and neither modification. More importantly, all the analyses run on the combined WT and MUT samples failed to identify "differential" states in which one of the two modifications was present only in WT or MUT samples. That is, the model built, regardless of the number of states given as input, consistently contained four more states corresponding to 1) the presence of both H3K27ac and H3K4me1 in both WT and MUT samples; 2) to H3K27ac in both WT and MUT samples; 3) to H3K4me1 in both WT and MUT samples; 4) to neither modification in WT or MUT samples.

Differentially enriched 200bp samples were identified with an approach similar to the one of ChromHMM, by comparing for each modification the number of mapped reads in the segments in the two WT samples to the number of reads of the two MUT samples. Given a region with *n* reads in WT and *m* reads for MUT, then we compute the probability of finding *n* and *m* reads by chance, given N mapped reads for WT and M for MUT, with a Chi-Square test. We considered "differentially enriched" all regions with the resulting p value lower than 10^{-4}.

Enrichment of SOX2-bound sites within H3K27Ac-enriched regions

Significance of overlap between H3K27ac-enriched regions and SOX2 binding sites was analyzed in comparison to 1,000 sets of random genomic DNA (random sampling). These sets comprised the same number of elements of similar size selected randomly from the mm9 genome excluding GAP regions (UCSC genome browser), blacklisted regions (cite PMID: 22955616) and unmappable regions. To define unmappable regions, bam-files from all H3K27ac and H3K4me1 ChIP-seq datasets (n = 8) were merged and mapped onto the mm9 genome that was binned in sliding windows of 3000 bp with an overlap of 500 bp. Bins with zero reads were defined as unmappable and thus excluded.

Zebrafish transgenesis

Sequences from 17 of the identified anchors (15 distal, and 2 proximal) were amplified from the mouse genome using specific primers (Table S1) and cloned into a pBluescript vector by a pCR8/GW/TOPO® TA Cloning® Kit (Life Technologies). Individual fragments were then transferred, using recombination-mediated, Clonase™-assisted, Gateway® technology (Invitrogen), to the ZED (Zebrafish Enhancer Detection) vector (Bessa et al., 2009). ZED contains a cardiac actin promoter-driven RFP gene, used as an internal transgenesis control, and a minimal promoter linked to the putative enhancer being tested, and driving GFP expression. Plasmid DNA was purified using the Genopure plasmid Midi kit (Roche) following manufacturer instructions. Zebrafish embryos were microinjected at one-cell stage with 3–5 nL of a solution containing 25 nM of each of the construct to be tested and 25 ng/ml of Tol2 RNA. Putative transgenic embryos, as determined by the expression of cardiac-actin:RFP, were screened for tissue-specific enhancer activity by looking for EGFP expression in the brain at 15, 18, 24 and 48 hpf stages. Fluorescent images were acquired with a black-and white highly-sensitive camera (Leica DC350FX) and converted into color images with the associated Leica acquisition program. EGFP distribution was compared with the expression pattern of the putative regulated genes, as determined by *in situ* hybridization analysis (from http://zfin.org). EGFP-positive (in forebrain) embryos were collected and propagated to generate three independent F1 transgenic lines each by crossing with wild-type animals. In the case of the en1 and en2 *c-fos* enhancer embryos were analyzed only at F0. To confirm a link between *Sox2* and the identified elements, embryos derived from the F1 lines were microinjected in one blastomere at 1-2 cell stage with a *sox2* specific morpholino (GeneTools; 50-500 nM), previously reported to efficiently interfere with *sox2* expression in zebrafish, without however causing major morphological defects (Okuda et al., 2010). To complement this study, the pCS2 plasmid containing the *sox2* coding sequence was linearized and *in vitro* transcribed using the SP6 MessagemMachine kit (Ambion). The synthesized mRNA (Esteve et al., 2004) was purified using Quiaquick RNeasy columns (Quiagen), precipitated, quantified and injected at the concentration of 100ng/ul into embryos derived from the F1 lines as above. Embryos were grown and scored for increased or reduced reporter expression at 15, 18, 24 and 48 hpf stages against the levels presented in untreated embryos or embryos injected with either a Genetools standard control MO or an unrelated (mCherry) mRNA, used as controls.

RNA-Seq analysis

RNA extraction was performed on three independent NSC populations for both wild-type and mutant cells, using Trizol and RNeasy Kit (QIAGEN). 1/5 volume of chloroform was added to one volume of Trizol. Aqueuos phase was transferred into a new tube. 1.5x volume of ethanol was added and mixed well. Mixture was was filtered through Rneasy (QIAGEN) column. Column was washed with Buffer RW1. On-column DNase treatment was performed as described by the RNase-Free DNase Kit (QIAGEN). Post treatment column was clean up with Buffer RW1 and two washes of RPE buffer. Column was then dried and total RNA was eluted with RNase-free water. PolyA Stranded Truseq Libraries were generated using the Truseq Stranded mRNA Sample Preparation Kit (Illumina). First, mRNA was purified from 1μg of total RNA using magnetic beads containing poly-T oligos. mRNA was then fragmented and reversed transcribed using Superscript II (Invitrogen), followed by second strand synthesis. Double stranded cDNA was treated with end-pair, A-tailing, adaptor ligation and 8 cycles of PCR amplification.

RNA-Seq was performed on triplicates for the two genotypes studied, yielding 51 bp single-end reads. The number of sequences obtained in each sample ranged from 7.5 to 12.5 millions.

Read counts and transcript levels for each sample were computed with the RSEM software package version 1.17 (Li and Dewey, 2011), on the RefSeq gene annotation available at the UCSC Genome Browser for mouse genome assembly mm9 (24,148 genes). For downstream analyses, expression levels measured as Transcripts per Million (TPM) were employed.

Expression boxplots and subsequent tests were generated using the R functions “boxplot” and “Wilcox.test.” Differential expression analysis was performed on the TPM values with the Noiseq package (Tarazona et al., 2015)(and refs. therein) using the NoiseqBIO method that handles replicate experiments. In this analysis, we considered to be having a significant variation of expression (Group 1) those genes with both 1) a fold ratio of the average transcript level in the two conditions greater than 1.5 and 2) an associated false discovery rate lower than 0.05 (corresponding to a Noiseq q-value greater than 0.95). As control for the analysis on co-associations between interacting anchors and differential expression, we also defined as having a “moderate” change of expression (Group 2) genes that did not satisfy either of the two previous conditions but had a FDR < 0.2 (q-value > 0.8), and “not changing” (Group 3) all the remaining genes with FDR > 0.2.

Co-association scores

Co-association scores are based on the significance of the overlap of two sets of genes, in this case one set showing a significant change of expression in the RNA-Seq data and a second one of genes whose promoter associated with a given type of interaction. Given two sets of genes of size n and m out of N annotated genes, and k genes in common to the two sets, the probability of having k genes in common by chance can be estimated by a Fisher’s exact test with parameters k (number of successes), n (size of the sample), m (number of successes on the population), N (size of the population). The test was applied by computing the number k of genes involved in any type of interaction (wTR1, wTR2, wTR3) in each of the three expression variation groups. Co-association scores were computed starting from the p value p resulting from the test, defined as –log10 p if k was greater than the expected value (hence showing a co-association between the two gene classes greater than what expected by chance), log10 p otherwise (hence showing a negative co-association). Thus, the higher positive co-association scores are, the more significant is the overlap between the two categories considered, with co-association scores greater than 2 showing a statistically significant overlap. Vice versa for negative scores, showing a significantly low overlap between two sets if lower than −2. The coassociation scores have been calculated using genes expressed at levels of at least 5 TPM in wt NSC; we also did the same analysis using all expressed genes: the results are qualitatively similar, and are not shown.

Socs3 transduction in NSC

The TWEEN lentiviral vector expressing SOCS3 from the CMV promoter and GFP driven by the human PGK promoter (Francipane et al., 2009) was transfected into low-passage 293T cells by calcium phosphate precipitation (overnight) together with the VSV-G plasmid (encoding ENV), CMV R8.74 (packaging) and pRSV-REV (encoding reverse transcriptase). Following replacement with fresh medium, the cell supernatants were collected at 24-48 hours from transfection. For NSC transduction, wild-type and *Sox2* mutant neurospheres (obtained as in Favaro et al., 2009) were grown for 2-3 passages in bFGF and EGF, and for one more passage in EGF only. They were then dissociated to single cells and seeded at a density of 25,000 cells/ 1ml/ well (9 wells for each cell type) in 24 well plates, in DMEM-F12 with Glutamax (GIBCO) containing EGF only as mitogen. After 4 hours wt and mut NSC were transduced with the *Socs3*-expressing vector at a multiplicity of infection (MOI) of 3.5-5.5 and incubated overnight at 37°C. Then 1ml per well of fresh medium was added both to transduced and non-transduced (control) cells. After 4 days, cells were dissociated to single cells, counted, and seeded at a density of 20,000 cell/well in the same EGF medium described above. In addition, 500,000 cells for each sample (from pooled wells) were fixed using PFA 4% and washed in PBS in order to analyze the GFP fluorescence by flow cytometry (BD FACSCalibur): 10,000 events were analyzed for each sample. The samples were excited at 488 nm (blue laser) and the resulting fluorescence measured at wavelengths > 530 nm. The results were analyzed using CellQuest Pro software (BD Biosciences).

DATA AND SOFTWARE AVAILABILITY

Accession numbers

The data discussed in this publication have been deposited in NCBI’s Gene Expression Omnibus (GEO). The accession number for the ChIA-PET, ChIPseq and RNAseq data reported in this paper is GEO: GSE90561

The genomic data can be visualized through the WashU browser:

http://epigenomegateway.wustl.edu/legacy/?genome=mm9&datahub=https://wangftp.wustl.edu/~dli/7131149234337a58201ae3da174ecc51/hub&coordinate=chr8:87120161-87587163

Cell Stem Cell, Volume *24*

Supplemental Information

Mapping the Global Chromatin Connectivity Network

for Sox2 Function in Neural Stem Cell Maintenance

Jessica A. Bertolini, Rebecca Favaro, Yanfen Zhu, Miriam Pagin, Chew Yee Ngan, Chee Hong Wong, Harianto Tjong, Marit W. Vermunt, Ben Martynoga, Cristiana Barone, Jessica Mariani, Marcos Julián Cardozo, Noemi Tabanera, Federico Zambelli, Sara Mercurio, Sergio Ottolenghi, Paul Robson, Menno P. Creyghton, Paola Bovolenta, Giulio Pavesi, Francois Guillemot, Silvia K. Nicolis, and Chia-Lin Wei

Supplemental Information

Figures S1-S6
Tables S2, S6, S7; Tables S1, S3, S4, S5, S8 are separate Excel files.

Figure S1, related to Figure 1
Overview of Chia-PET and in situ ChIA-PET workflow

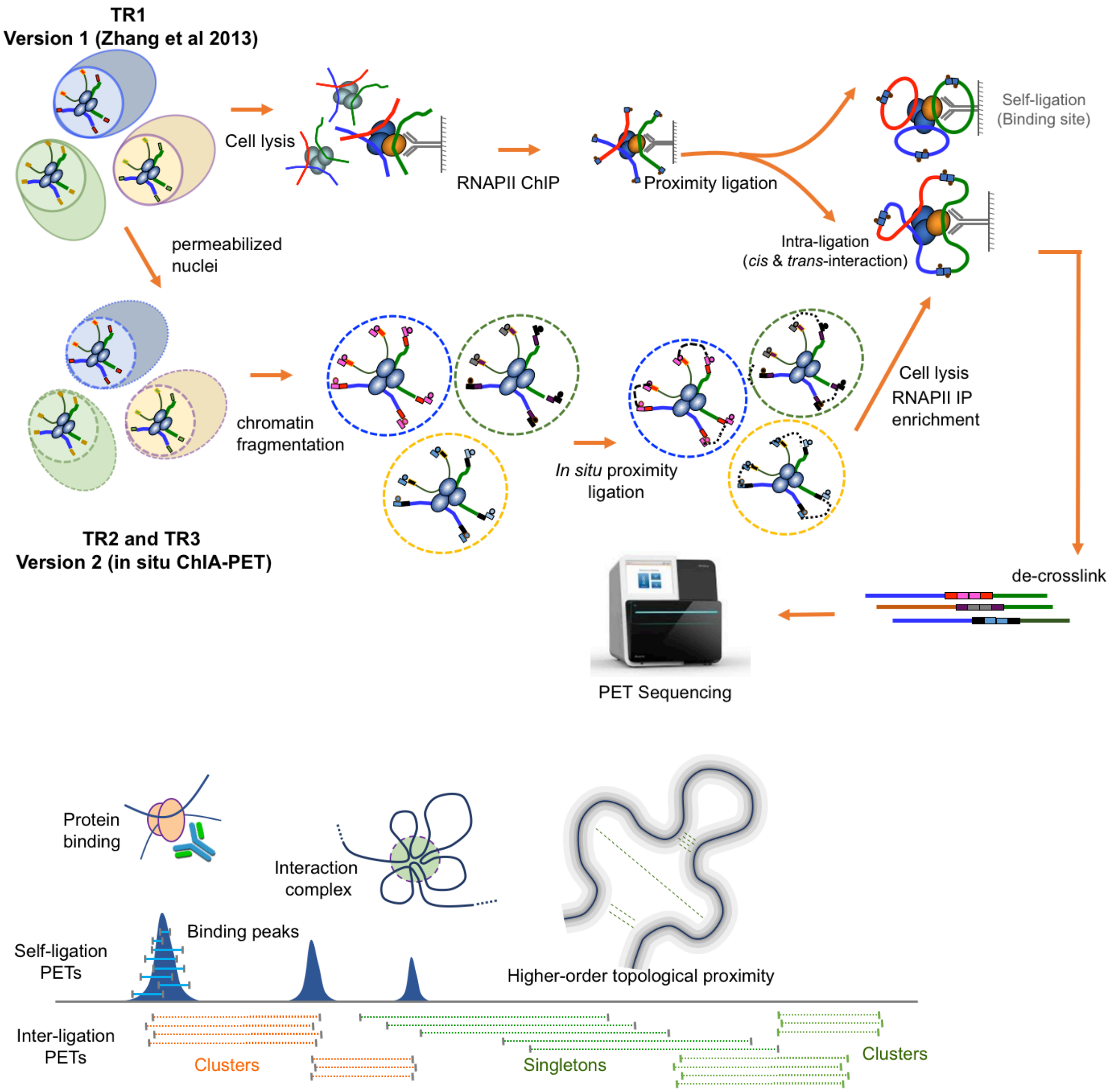

ChIA-PET was as previously described by Zhang et al., 2013; after crosslinking, all additional procedures were performed after cell lysis. For in situ ChIA-PET, the nuclei of the crosslinked cells were permeabilized when still intact, allowing to process chromatin for proximity ligation prior to cell lysis (see Methods).
In the bottom panel, different types of inter-ligation PETs are shown. In addition to revealing RNAPolII-mediated interactions, in situ ChIA-PET may reveal, when considering the entire set of intra-molecular ligated PETs (bottom panel) also general chromatin contacts that represent broad spatial topological associated domains, similar to the global contact maps generated by Hi-C based approaches (data not shown).

Figure S2, related to Figure 1
Examples of reproducibility in different biological replicates

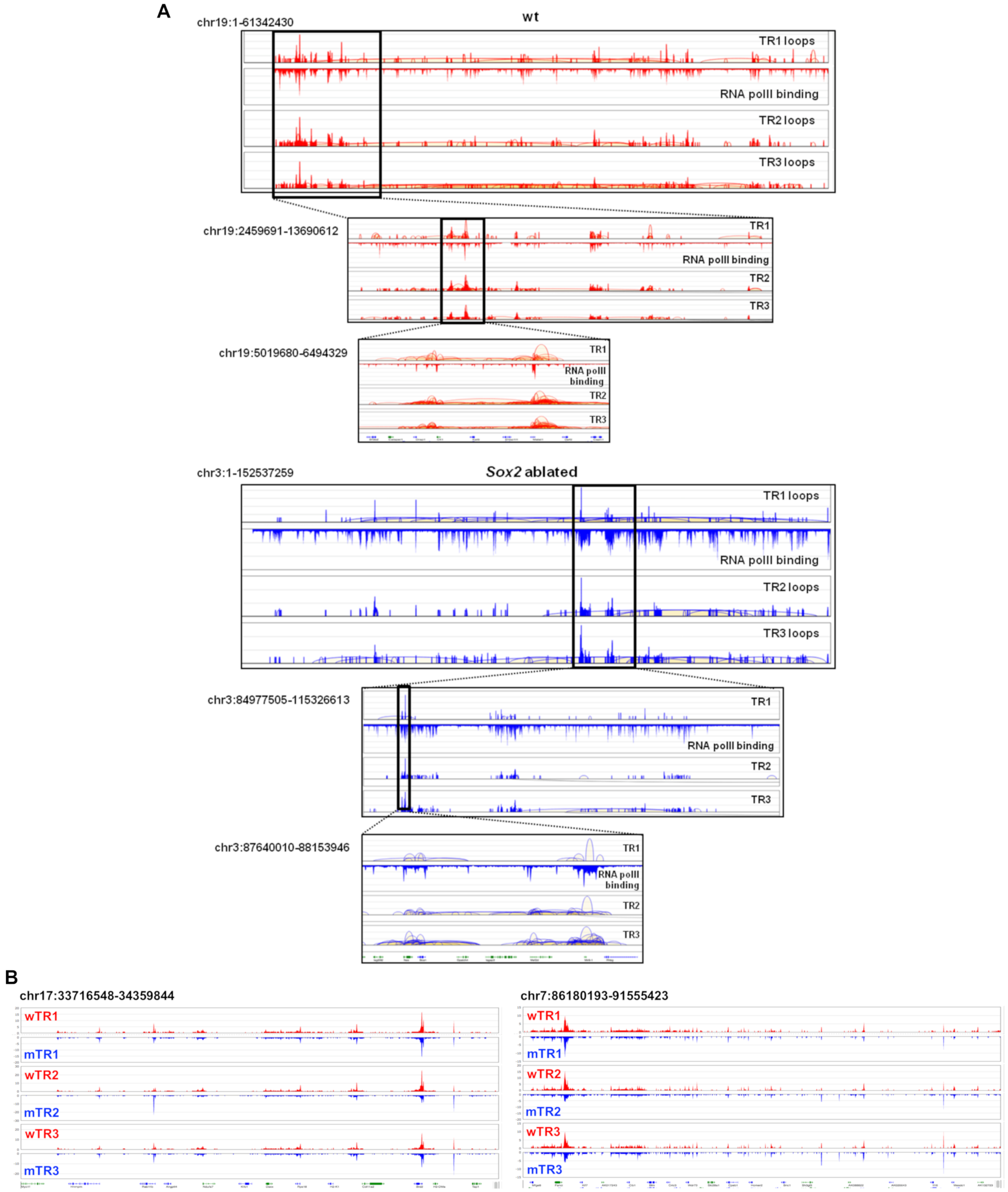

(A) Examples of interactions (loops) profiles in TR1, TR3, TR3, in wild-type cells (red, top) and mutant cells (blue, bottom), in the indicated chromosome regions. The boxed regions are zoomed-in to allow observation at higher resolution.

(B) Examples of RNApolII binding profiles in wild type and mutant TR1, TR2 and TR3 samples.

Figure S3, related to Figure 1
Comparison of PET counts between wild-type and Sox2-mutant libraries (TR2 and TR3) across 9 view points

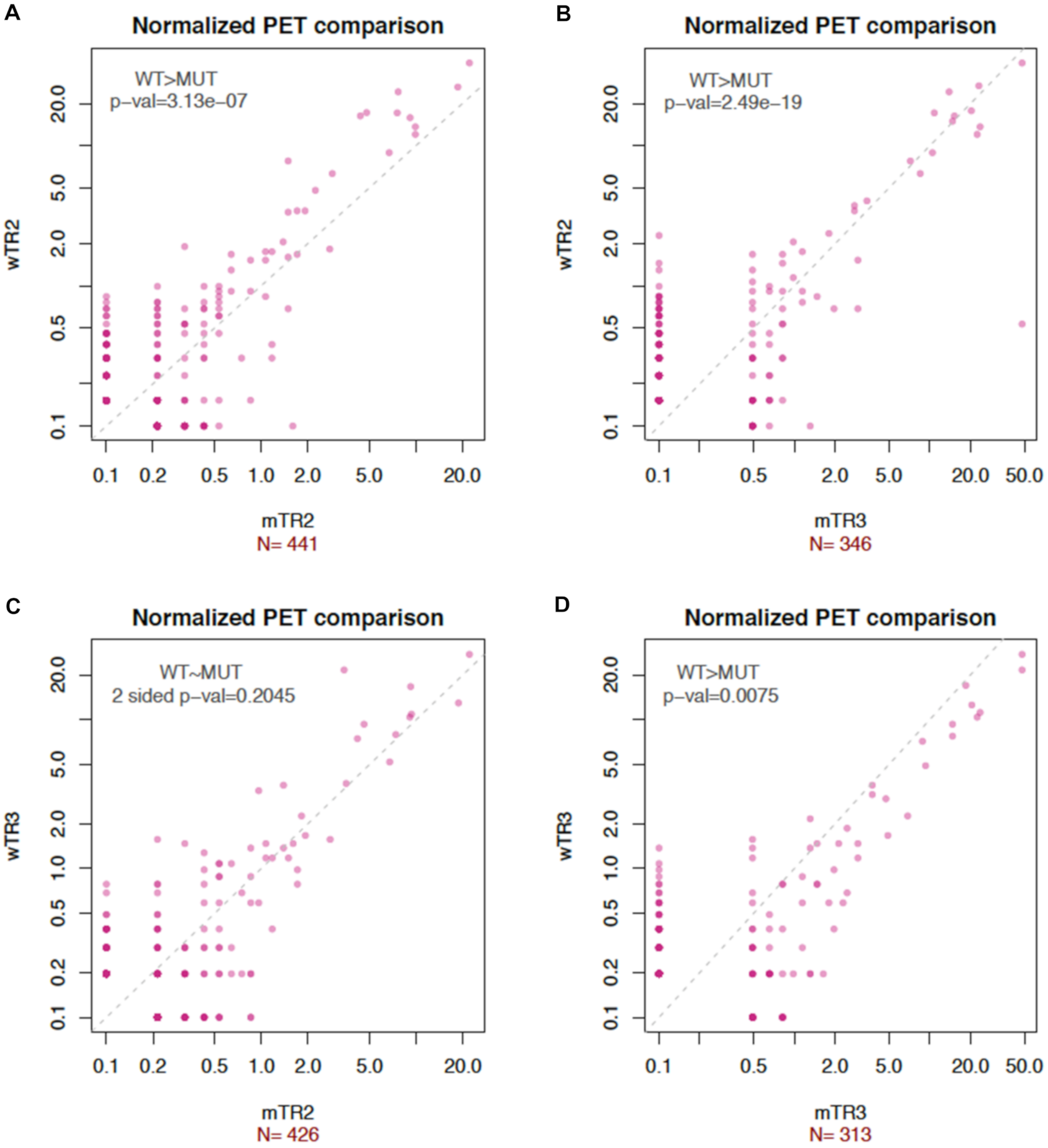

Comparisons are:
A) between wtTR2 and mtTR2; B) between wtTR2 and mtTR3;
C) between wtTR3 and mtTR2; D) between wtTR3 and mtTR3. All cis-interactions along the chromosome from compared libraries that originated from all view points listed in the Table below (+/- 5kb) were collected. The interaction anchors were then evaluated to assess whether they overlap. As a result, a new set of non-overlapping interactions was then collected (see the Table for number of interactions or dots on each panel). When an interaction was absent in one of the two libraries, its PET count was assigned as 0. The compared data set is compiled from the output of a significance-calling algorithm, using down-sampled data (Table 1) to minimize sequencing depth bias.

The PET counts for each interaction (normalized by dividing by the total number of intrachromosomal PETs -Table 1, line 6- , then multiplying by one million) are plotted as dots, with WT PET count on the Y axis, and MUT PET count on the X axis; the dashed line represents equal interaction frequency, so points above this line indicate reduction of interaction frequency in MUT. Using log-log scale plotting, the zero PET counts are all augmented by 0.1. Because the dots are transparent, the color intensity of the dots indicates multiple dots having the same WT/MUT PET counts. We statistically evaluated differences between wild type and mutant; differences for MUT < WT are shown as p-values on the plot area (2-sided Wilcoxon rank sum test p-values). mTR3 was highly significantly reduced relative to wTR2 and wTR3; mTR2 was less significantly reduced. The comparison of WT and MUT replicates between themselves yielded non-significant p-values (0.029 and 0.09039, respectively). For this analysis, the threshold for interaction calling was set to PET count = 2, FDR < 0,05 across all 9 loci, yielding a number of interactions adequate for statistical analysis. N: the total number of interactions (dots on the plots) evaluated for each comparison.
The numbers of PET counts are listed in Table S3.

For each viewpoint, the genomic coordinates are given. Number of interactions (dots) are shown on the right for each viewpoint, and each panel.

	**Viewpoint**	**CHR**	**Mid_Coordinate**	**panel A**	**panel B**	**panel C**	**panel D**
1	c-Fos-enh5	chr12	86,797,329	43	36	39	32
2	c-Fos-prom	chr12	86,814,992	63	53	70	58
3	c-Fos_enh3	chr12	86,829,043	54	47	38	29
4	Sox3_prom	chrX	58,145,388	10	7	11	8
5	Olig1_prom	chr16	91,270,745	66	54	74	53
6	Olig2_prom	chr16	91,224,410	102	80	93	70
7	Kat2b_prom	chr17	53,707,872	24	20	20	13
8	Tcf4_prom	chr18	69,505,556	29	10	34	17
9	Stox2_prom	chr8	48,372,611	50	39	47	33
**Total**				**441**	**346**	**426**	**313**

Figure S4, related to Figure 2

Long-range interaction anchors are enriched in SOX2 binding and in chromatin epigenetic marks of active/poised enhancers

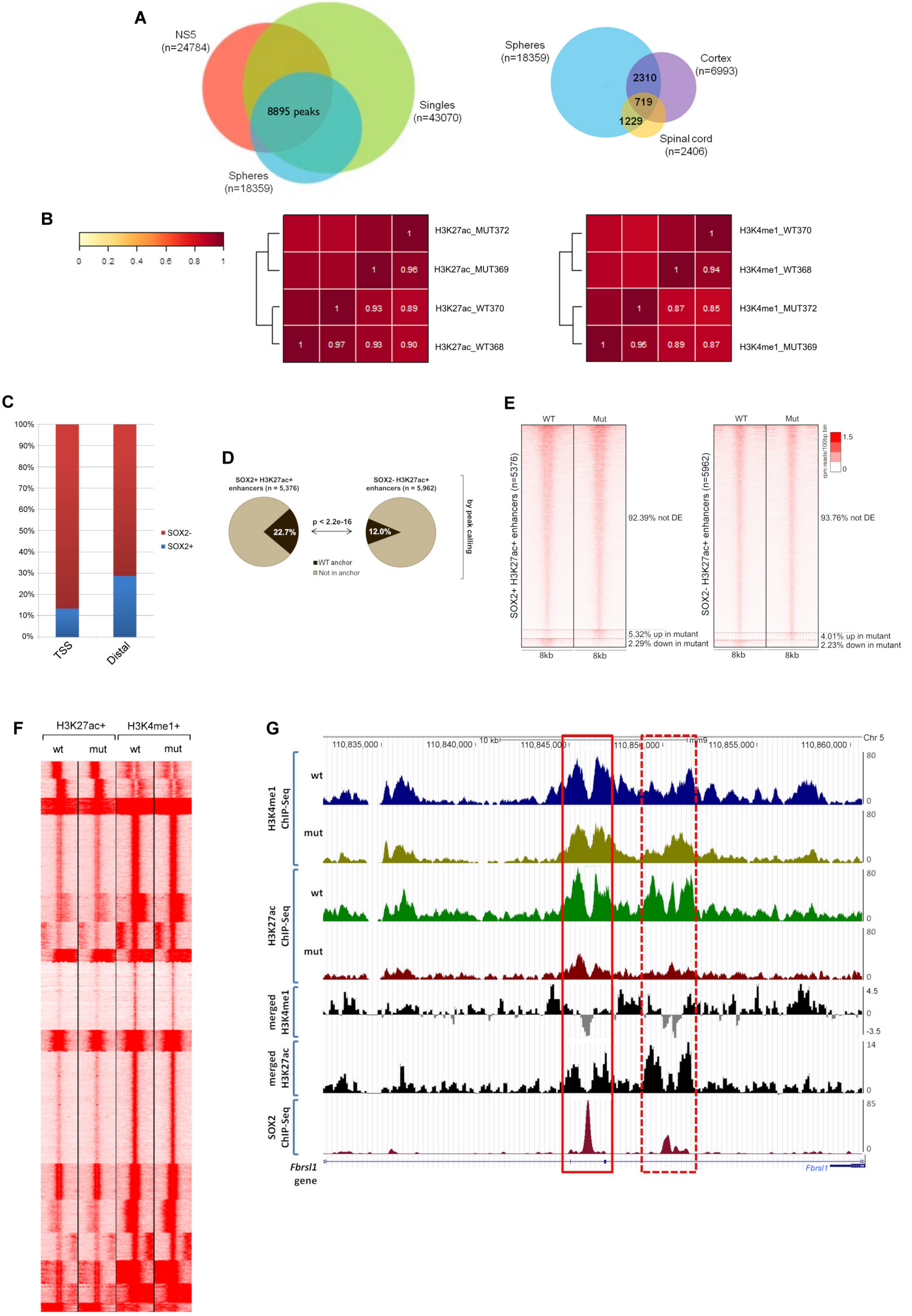

(A) SOX2 ChIPseq was performed on both intact ("spheres") and dissociated ("singles") neurospheres (see Methods). Ca. 87% of peaks identified in spheres are also present in singles; ca. 50% of peaks in spheres overlap with peaks in the ES cell-derived NS-5 neural stem cell line (Mateo et al., 2015). Ca. 33% and 51% of peaks detected in neural progenitors of cortical and spinal cord origin, and 68% of their intersection (Hagey et al., 2016), are also detected in spheres.
(B) Pearson correlations between the samples for each antibody used in duplicate samples.
(C) Fraction of SOX2+ and SOX2- regions within epigenetically marked regions (i.e. H3K27ac+ and/or H3K4me1+) (ChromHMM).
(D) Fraction of Sox2+ vs. Sox2- EM-positive regions which overlap with anchors. Distal (>1000nt from TSS) Ac+ regions, peak-calling.
(E) *Sox2* loss does not result in H3K27ac-enrichment changes at enhancers. Heatmap depicts H3K27ac enrichment (for one wt and one mutant line) for SOX2-positive and SOX2-negative enhancers. Reads were counted within 40 bins of 100 bps up- and downstream of the enhancer centre. The fraction of (not) significantly differentially enriched enhancers, as defined using DESeq2, is indicated.
(F) *Sox2* loss does not result in H3K27ac- or H3K4me1-enrichment changes at regions bound by SOX2 in wt cells.
(G) A representative example of quantitative differences in epigenetic enhancer marks between wt and mut cells (SOX2 peaks within the *Fbrsl1*/*AUTS2l* gene, boxed in red). Merged profiles represent the variation of enrichment, positive values correspond to greater enrichment in wt, negative in mut, respectively. Typically, H3K27ac has two peaks flanking the SOX2 binding site: the height of these peaks is decreased in mutant cells, whereas H3K4me1 has a moderate increase in mut closer to SOX2 binding sites, as also shown for the second SOX2 peak on the right, where similar changes occur.

Figure S5, related to Fig. 3

Enrichment of VISTA enhancers within interaction anchors

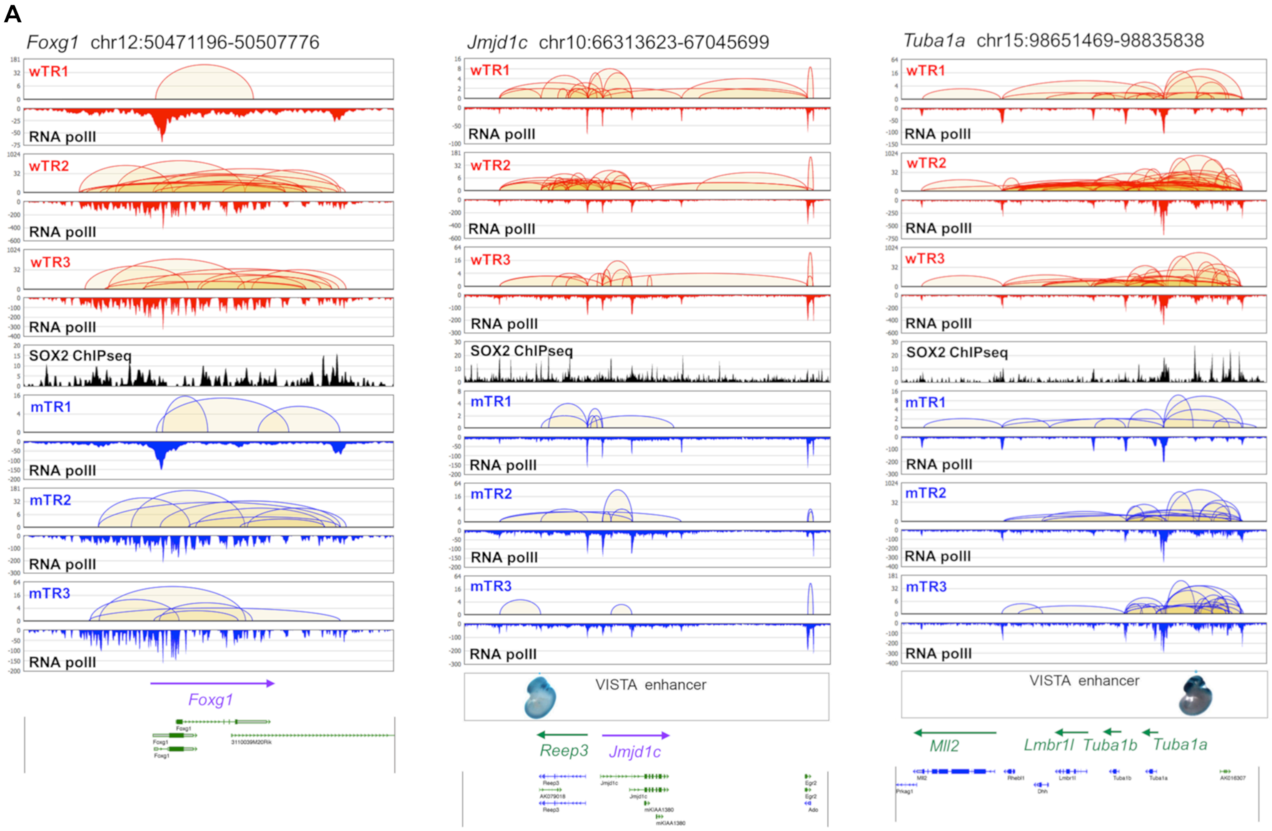

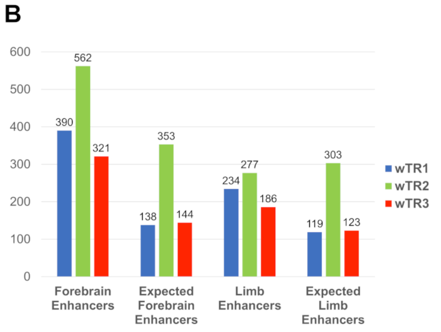

Forebrain p300 enhancers	Total number	2454

	VISTA enhancers in all anchors	VISTA enhancers in distal anchors	% Overlap of VISTA enhancers with anchors	% Distal overlap*	No. of anchors	No. of distal anchors	% of distal anchors	Expected No. of overlapping VISTA enhancers in anchors
wTR1	390	256	15.89	65.64	14138	4639	32.81	138
wTR2	562	405	22.90	72.06	36012	13435	37.31	353
wTR3	321	211	13.08	65.73	14680	5005	34.09	144
mTR1	104	31	4.24	29.81	6488	2404	37.05	63
mTR2	105	33	4.28	31.43	5742	1681	29.28	56
mTR3	99	35	4.03	35.35	6402	2234	34.90	62

* % distal versus total VISTA enhancers overlapping with anchors

(A) Examples of replicates of ChIA-PET analysis of regions surrounding genes encoding proteins important for inherited brain developmental defects (See also **Table S7**). Images (from https://enhancer.lbl.gov/) of embryos carrying lacZ transgenes driven by VISTA enhancers located within the considered genomic regions are shown.

(B) Left: Numbers of VISTA enhancers from forebrain or limb (forebrain enhancers, limb enhancers; Visel et al., 2009) in interaction anchors, as compared to the numbers expected based on a random distribution. Limb enhancers are presented as a term of comparison between regulatory elements active in forebrain neural tissue versus non-neural tissue.

Right: Overlap of forebrain VISTA enhancers with anchor types.

Figure S6, related to Figures 3 and 4
Regulation of the Sp8 enhancer-dependent GFP-reporter construct by decreased or increased Sox2 expression

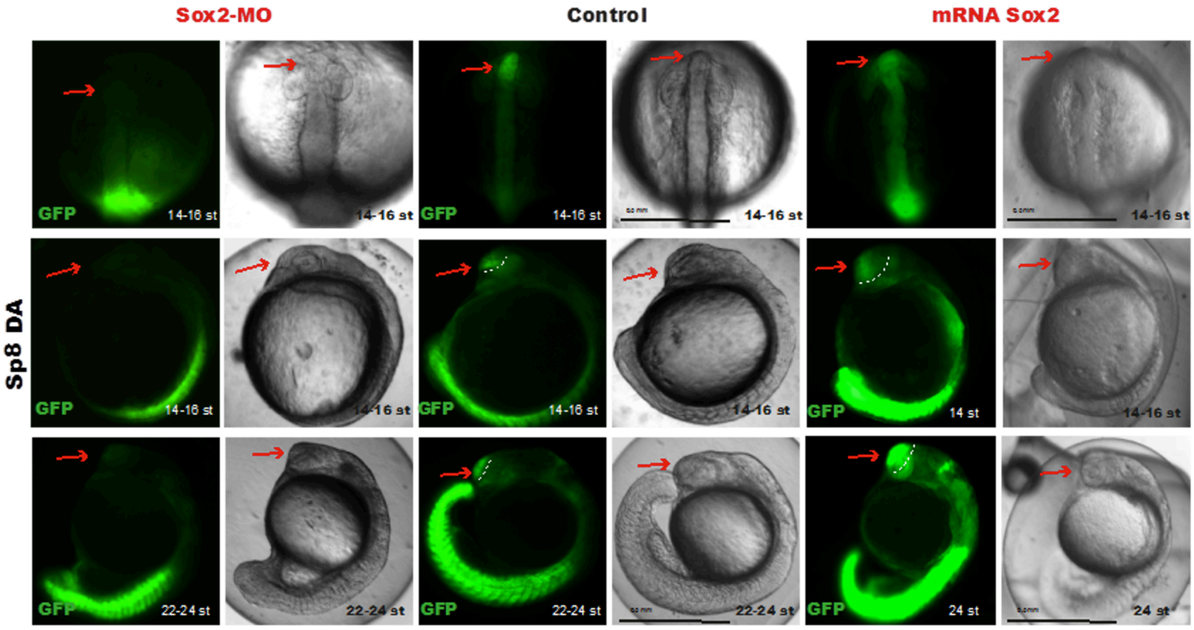

Transgenic embryos injected with anti-Sox2 morpholino (*Sox2*-MO, left), control morpholino (center), Sox2mRNA and analyzed at two different stages. Reduced GFP signal is seen in *Sox2*-MO, but not ctrl-MO embryos, in forebrain (red arrow) but not in more posterior regions (internal control). Sox2 mRNA extends the forebrain expression of the transgene (in 19/54 embryos). Top, dorsal view; middle and bottom, lateral view.

Tables S2, S6, S7

(Excel Supplemental Tables are provided separately)

Table S2, related to Figure 1
Average reproducibility score (SCC, Stratum-adjusted Correlation Coefficient*) between TR2 & TR3 over all 20 chromosomes
WT: 0.935 (wTR2 vs. wTR3).
MUT: 0.839 (mutTR2 vs. mutTR3).

SCC break down by chromosome between replicas:

**Chrom**	WT	MUT
chr1	0.938	0.846
chr2	0.918	0.813
chr3	0.935	0.819
chr4	0.946	0.859
chr5	0.920	0.826
chr6	0.920	0.815
chr7	0.944	0.857
chr8	0.936	0.854
chr9	0.930	0.827
chr10	0.925	0.813
chr11	0.924	0.823
chr12	0.946	0.866
chr13	0.964	0.877
chr14	0.951	0.867
chr15	0.933	0.849
chr16	0.941	0.850
chr17	0.939	0.862
chr18	0.944	0.855
chr19	0.942	0.855
chrX	0.905	0.755

Method:
To compute the SCC, ChiA-PET loops for each library were aggregated into 10-kb bin matrix.
SCC score is computed with HiCrep tool (hicrep library in R; Yang et al. (2017) Genome Research 27:1939).
For each chromosome, the smoothing parameter used as recommended (h=5) and maximum distance 1 Mb.

*reference: https://genome.cshlp.org/content/early/2017/08/30/gr.220640.117
Pearson's correlation coefficient (PCC) is computed for loops within the same genomic distance. From all distances considered, the PCCs are then weight averaged.

Table S6, related to Figure 3, Fig. S5 and Table S5
Interactions and SOX2 peaks in mouse homologs of genes involved in inherited neural disease in man

Category of disease and gene name in mouse	Disease gene promoter interaction with distal enhancer: wt-sp	wt-alt	com	SOX2-bound enh.	SOX2-bound
**Microcephaly associated to defects in**					
**centrosome and spindle microtubule (1)**					
positive: 10/22; p-value: 0.006					
Cdk5rap2 (Mcph3)					●
Casc5					●
Cenpj					●
Stil					●
Cep63		●		●	
Kif2a		●			
Kif11	●				
Tubb2b		●	●	●	●
Tuba1a		●	●	●	●
Poc1a					●
**origin recognition complex core (1)**					
positive: 2/5					
Orc4					●
Cdt1					●
**DNA damage response and repair (1)**					
positive: 5/19					
Lig4					●
Phc1 (Mcph11)		●		●	●
Xrcc2					●
Xrcc4					●
Blm (Recql3)					●
**Other microcephalies**					
Gpr56		●	●	●	●
Cdk19					●
Arx			●		
Zbtb18					●
**Angelman and Angelman-like syndromes (2)**					
**(intellectual disability and absent speech)**					
positive: 10/12; p-value: 0.0000065					
Ube3a					●
Tcf4	●	●	●	●	●
Ehmt1					●
Herc2		●		●	
Adsl					●
Cdkl5					●
MeCP2					●
Foxg1		●		●	
Atrx					●
Zeb2	●	●		●	●

Category of disease and gene name in mouse	Disease gene promoter interaction with distal enhancer: wt-sp	wt-alt	com	SOX2-bound enh.	SOX2-bound
**Histone modification, chromatin remodelling**					
**and mediator mutations (3,4,5)**					
**(intellectual disability)**					
positive: 8/12; p-value: 0.0007					
Med17					●
Med23					●
Med25					●
Smarca2					●
Arid1a		●	●	●	
Arid1b		●	●		
Jmjd1c		●	●	●	
Phf21a					●
**Cohesin subunit mutations (6)**					
**(psicomotor delay, intellectual disability)**					
positive: 4/14					
Smc3					●
Rad21 (Scc1)					●
Stag1		●		●	
Stag2		●			
**Microphthalmia / Anopththalmia /**					
**Coloboma and other eye pathologies (7)**					
Otx2		●			●
Pax6					●
Six3					●
Bmp7	●			●	
Grcc10 (C12orf57)		●			●
Sall2					●
RarB	●				●
Smoc1	●			●	
Wdr19					●
COUP-TF1 (Nr2f1)	●			●	
Abhd12	●			●	

References:
(1) Alcantara and O'Driscoll, 2014, Am J Med Genet C Semin Med Genet 166C, 124
(2) Tan et al., 2014, Am J Med Genet A 164°, 975-992
(3) Lee and Young, 2013, Cell 152, 1237-1251
(4) Saez et al., 2016, Genetics in Medicine 18, 378-385
(5) Kim et al., 2012, Am J Hum Genet 91, 56-72
(6) Peters et al., 2008, Genes Dev 22, 3089-3114
(7) Williamson and Fitzpatrick, 2014, Eur J Med Genet 57, 369-380

We list genes related to the main categories of neurodevelopmental disorders in man that are either present in SOX2-mutant patients (hippocampal, eye defects; intellectual disability, seizures) and/or in Sox2-mutant mice (microcephaly, hippocampal defects, seizures, eye defects). Disease genes are analyzed for interactions of the respective mouse gene promoter with distal enhancers in wTR1, or presence of SOX2. For replicates (wTR2 and wTR3) of connectivity analyses for some of these genes see Fig. 3 and Fig. S5. p-values are given, when significant, for the association between the above characteristics and type of disease.

Table S7, related to Figure 4
Summary of transgenic experiments in zebrafish

Gene associated with the proximal anchor	Cloned anchor [DA: distal anchor; PA: proximal anchor]	Distance PA-DA [bp]	Presence of SOX2 peaks (ChIP-seq)				Presence of already defined forebrain enhancer (VISTA enhancer)		Tested in fish (GFP+ at 24 hpf)				
			cloned anchor		associated anchor		p300 binding site	validated enhancer	reproducible GFP+ expression in forebrain	GFP+ in forebrain / tot transgenics		GFP+ in forebrain and elsewhere / tot transgenics	intensity of GFP+ expression in forebrain
			in our NSCs	in other ChIP-seq (10)	in our NSCs	in other ChIP-seq (10)				%	n°	n°	
**Nkx2.1**	DA504	10,000	-	✓	-	-	✓	✓	+	94.7%	36/38	37/38	++++
**Sp8**	PA545	10,000	-	-	✓	✓	-	-	+	75.0%	15/20	15/20	+
	DA545		✓	✓	-	-	-	-	+	94.9%	37/39 (1)	39/39	++++
**Coup-TF1**	DA2467	12,000	✓	-	✓	✓	-	-	+	35.0%	7/20	7/20	+
	DA602	27,000	✓	-	-	-	-	-	+	40.0%	8/20	8/20	+
**Ntng1**	DA1414	100,000	✓(2)	✓	✓(2)	-	-	-	+/-	2.6%	(1-3)/39 (3)	(5-7)/39 (4)	+
**Irx1**	DA597	450,000	✓	✓	-	✓	✓	(6)	+	49.1%	29/59	53/59 (5)	++
**Socs3**	DA463	32,000	✓	✓	✓	✓	✓	(6)	+	23.5%	8/34 (7)	16/34	+
**Chd7**	DA1439	40,000	✓	✓	-	-	-	-	+	13.6%	3/22	9/22	++
**Sox3**	DA2733	350,000	-	- (8)	-	-	✓	✓	+	100.0%	130/130	130/130	+++++
	DA2702	65,000	✓	✓	-	-	-	-	+	81.2%	13/16	15/16	+++
**Sox4**	DA2458	650,000	✓	✓	✓	✓	✓	✓	+	93.4%	114/122	119/122	+++
**Cxcr4**	DA62	180,000	✓	✓	-	-	✓	(6)	+	31.2%	30/96	56/96	++
**Zfp335**	DA1303	125,000	✓	-	-	✓	-	-	-	0%	0/56	4/56	-
	PA1303		-	✓	✓	-	-	-	-	0%	0/25	5/25	-
**c-Fos**	DA852 (e1)	40,000	✓	-	✓	✓	✓	✓	+	22.2%	12/54	54/54 (9)	+++
	DA854 (e2)	20,000	✓	✓	✓	-	✓	✓	+	9.6%	7/73	73/73 (9)	+

(1): 10/37 GFP+ also in midbrain-hindbrain boundary;
(2): the peak is not completely included;
(3): one embryo with clearly positive signal, two with weak signal;
(4): five embryos with clearly positive signal, two with weak signal;
(5): 18/53 in more posterior neural tube regions;
(6): not present in VISTA enhancer;
(7): 2 embryos with clearly positive signal, 6 with weak signal;
(8): presence of a SOX3 peak;
(9): all transgenics showed some expression in the trunk;
(10): Lodato *et al.*, 2013; Engelen *et al.*, 2011.
